# Vapor‐Phase Grain‐Boundary Anchoring Enables Molecular Toughening and Record Bending Endurance in Pilot‐Scale Roll‐to‐Roll‐Printed Flexible Perovskite Modules

**DOI:** 10.1002/anie.7292679

**Published:** 2026-05-26

**Authors:** Lirong Dong, Viktor Rehm, Shudi Qiu, Zexian Han, Naveen Harindu Hemasiri, Michael Wurmshuber, Michael Wagner, Cindy‐Ly Tavera‐Méndez, Dorothea Wisser, Chaohui Li, Robin Basu, Leopold Lahn, Olga Kasian, Sarmad Feroze, Wolfgang Heiss, Andreas Distler, Hans‐Joachim Egelhaaf, Fu Yang, Christoph J. Brabec

**Affiliations:** ^1^ Institute of Materials for Electronics and Energy Technology (i‐MEET) Friedrich‐Alexander‐Universität Erlangen‐Nürnberg Erlangen Germany; ^2^ Institute of Materials for Electronics and Energy Technology (i‐MEET), Energy‐Campus‐Nürnberg Friedrich‐Alexander‐Universität Erlangen‐Nürnberg Nürnberg Germany; ^3^ Helmholtz Institute Erlangen‐Nürnberg For Renewable Energy (HI ERN), Institute for Photvoltaics (IMD‐3) Forschungszentrum Jülich GmbH Immerwahrstrasse 2 Erlangen Germany; ^4^ Institute of General Materials Properties Friedrich‐Alexander‐Universität Erlangen‐Nürnberg Erlangen Germany; ^5^ Erlangen Center for Interface Research and Catalysis Friedrich‐Alexander‐Universität Erlangen‐Nürnberg Erlangen Germany; ^6^ Helmholtz Zentrum Berlin GmbH Berlin Germany; ^7^ FAU Profile Center Solar Friedrich‐Alexander University Erlangen–Nürnberg Erlangen Germany; ^8^ College of Chemistry Chemical Engineering and Materials Science Soochow University Suzhou China; ^9^ Suzhou Sunflex New Energy Company Limited Suzhou China

## Abstract

Flexible lead‐halide perovskite solar modules with carbon electrodes (C‐fPSMs) are promising for printed photovoltaics due to their low cost and compatibility with roll‐to‐roll (R2R) fabrication. However, the mechanical fragility of microcrystalline perovskite films, arising from their high grain‐boundary density, limits their practical application. Here, we report a vapor‐phase grain‐boundary anchoring strategy via thiol vapor annealing to molecularly toughen printed perovskite layers. Thiol molecules interact with intermediate species and preferentially anchor at surfaces and grain boundaries, reprogramming crystallization across the film. This chemomechanical conditioning promotes grain growth, reduces trap density, and transforms the deformation behavior from brittle fracture to ductile strain accommodation, as evidenced by reduced Young's modulus, increased yield strain, and significantly enhanced fracture toughness. As a result, treated devices retain 93% of their initial performance after 25 000 bending cycles, representing a new benchmark for flexible perovskite cells. In addition, improved film uniformity and interfacial contact enable power conversion efficiencies of 15.7% for R2R‐printed cells and 12.1% for modules (20.25 cm^2^), with scalability demonstrated up to 900 cm^2^ (17.26%). This work provides a scalable strategy to simultaneously enhance mechanical robustness and photovoltaic performance.

## Graphical abstract

A vapor‐phase molecular toughening strategy is developed for pilot‐scale roll‐to‐roll (R2R) ‐compatible fabrication of flexible perovskite solar modules. Thiol vapor selectively regulates crystallization and anchors at grain boundaries, reducing defects while enhancing mechanical resilience. The treated perovskite films exhibit suppressed crack formation, improved fracture toughness, and enhanced operational durability, enabling highly bendable and scalable printed perovskite photovoltaics.

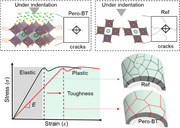

## 1 Introduction

Flexible lead‐halide‐perovskite solar modules with carbon electrodes (C‐fPSM) have introduced significant interest within the community focused on scaling up printable flexible photovoltaics (PV). Alongside the campaign for higher power conversion efficiency (PCE) on a laboratory scale, developing scalable approaches for the commercialization of PV based on perovskite has become a primary research focus. Achieving this goal involves a critical milestone: the development of a scalable production process for flexible perovskite modules that mitigates high capital expenditure, costly materials, slow processing rates, and low yields [1, 2]. The cornerstone of roll‐to‐roll (R2R) production is arguably the successful development of reliable processing protocols for printable PV [3, 4, 5, 6].

However, C‐fPSM produced via industrial R2R techniques experience inferior performance and compromised mechanical durability compared to the lab‐scale depositions [7, 8]. Specifically, the conditions for initiating perovskite crystallization are notably less efficient compared to traditional lab‐based techniques like spin‐coating. First reports have documented that deficient nucleation and insufficient crystal growth, which ultimately resulted in the formation of smaller, less‐ordered crystals with substantial lattice strain [9, 10, 11]. When the perovskite film has small grains with extensive grain boundaries, these boundaries act as stress concentrators that promote crack initiation. This microstructural characteristic leads to a brittle behavior where the film lacks the ability to deform plastically, resulting in an increased likelihood of crack formation and defects during R2R printing [10].

The resulting brittle fracture behavior is particularly detrimental for flexible modules, where repeated bending and processing‐induced tensile stress can readily generate microcracks, interfacial debonding, and electrically inactive regions, ultimately lowering yield and long‐term reliability. Therefore, a key unmet need in R2R‐printed flexible perovskite modules is a simple, scalable strategy that suppresses crack formation by simultaneously promoting grain growth, reducing grain‐boundary fragility, and enabling strain accommodation—without sacrificing compatibility with continuous manufacturing.

Another key aspect of the fully printable photovoltaics, the top electrode, which is essential in the device architecture, is still predominantly fabricated by thermally evaporating noble metals. However, to exploit the full potential of R2R production, it is desirable to eliminate all vacuum‐based processes, such as thermal evaporation. In our effort to use less costly and more stable materials, e.g. carbon black has proven highly promising as a replacement for the top metal electrode, as demonstrated in our previous work [12, 13, 14, 15].

Here we develop a vapor‐phase grain‐boundary anchoring (VP‐GBA) strategy enabled by thiol vapor annealing to molecularly toughen R2R‐printed perovskite films. Implemented on a pilot‐scale R2R coater, VP‐GBA produces larger perovskite grains with enhanced strain tolerance, yielding an order‐of‐magnitude increase in fracture toughness and markedly suppressed crack formation in printed perovskite films. We find that this approach results in larger perovskite grain sizes with increased yield strain, leading to an order‐of‐magnitude increase in fracture toughness in the printed perovskite film. This enhancement in the elastic resilience and fracture toughness results in enforced mechanical durability under scalable industrial production. Tailoring the mechanical properties of the perovskite layer is a win–win strategy for managing stress‐induced crack‐formation during R2R processing and improving device performance. As a result, we have achieved a record PCE of 12.1% in the reported R2R‐printed C‐fPSM, along with a PCE of 10.1% for a 100 cm^2^ large module. It additionally demonstrates exceptional mechanical stability by retaining 93% of its initial PCE after 25 000 bending cycles at a 5 mm curvature for carbon‐based solar cells. The method is further validated in inverted flexible modules, achieving a 17.26% PCE on a 900 cm^2^ device.

## 2 Results and Discussions

**FIGURE 1:**
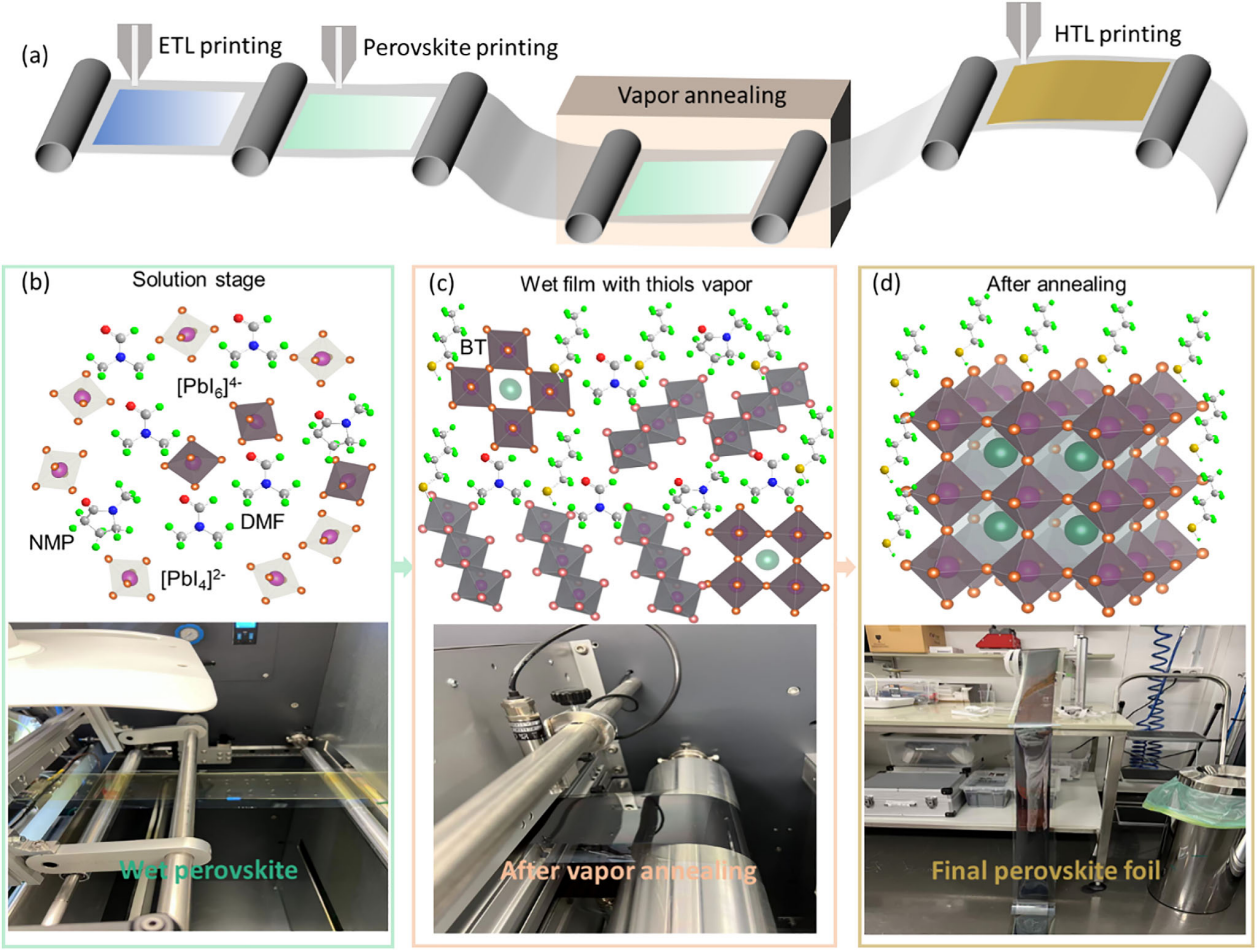
(a) The R2R production workflow includes the slot‐die coating of the ETL, perovskite deposition with thiol vapor treatments, followed by HTL and carbon electrode (for details, see the experimental section in SI). (b) The schematics of solution stage in as‐printed wet perovskite film after gas quenching. (c) The potential binding interactions of thiol within the perovskite during vapor annealing and corresponding perovskite film after vapor annealing. (d) The photograph of R2R printed perovskite with HTL on SnO_2_/IMI substrate.

Flexible perovskite solar cells and modules are produced via high‐throughput processing on polyethylene terephthalate (PET)/ITO‐Silver‐ITO (IMI) flexible substrates, using the layer stack PET/IMI/SnO_2_/perovskite/hole transporting layer (HTL)/Carbon, as illustrated in the schematic R2R production workflow, Figure 1a. The photograph of the R2R infrastructure can be found in Figure S1. The SnO_2_ layer is first applied to the patterned substrate using slot‐die coating on a R2R processing line. Subsequently, the IMI/SnO_2_‐substrate is overcoated with the perovskite layer, HTL, and top carbon electrode. This entire process occurs in ambient air (20% RH) and at low temperatures (≤ 120°C). The VP‐GBA strategy specifically involves transferring the gas‐quenched wet perovskite film to an integrated oven pre‐filled with thiol molecule vapor. As shown in Figure S2, a small volume (∼100 µL per sub‐oven) of thiol is pre‐deposited into the preheated oven to generate a low‐concentration vapor atmosphere (∼1.14 × 10^−4^ vol%) prior to the introduction of the foils. This concentration is well below the lowest explosive limit (LEL) of thiols, as summarized in Table S1, indicating a reduced flammability risk under the applied conditions. It should be noted, however, that the LEL criterion pertains only to ignition hazard and does not imply overall safety with respect to operator exposure or toxicity; therefore, appropriate engineering controls and safety precautions remain essential during operation.

We first screened a series of 1‐butanethiol (BT) and its isomers, including 2‐methyl‐1‐propanethiol (2M1PT) and 2‐methyl‐2‐propanethiol (2M2PT) and investigate their effect on the perovskite solar cells. In Figure S3, the photovoltaic performance of solar cells treated with different thiols is compared. It clearly demonstrates that the linear BT molecules deliver the best functionality and overall performance, whereas the branched isomers 2M2PT and 2M1PT are less effective. Additionally, Figure S4 shows top‐view scanning electron microscopy (SEM) images of perovskite films treated with different thiol vapors and reveals that BT can enable larger, more uniform grains with a significant reduction of residual PbI_2_ on the surface.

In the following part, we refer to the BT‐treated perovskite film as Pero‐BT in the following sections and focus our study on the mechanisms. We first systematically evaluated the impact of introducing BT at different stages of perovskite film formation. Among the three stages—precursor doping—solid‐state vapor annealing and intermediate‐phase vapor treatment—the last proved most effective, as demonstrated in Figure S5. Because BT is nonpolar and poorly soluble in polar aprotic solvents (e.g., DMF/DMSO), adding it to the precursor induces phase separation, while post‐crystallization vapor annealing provides only surface‐limited passivation. Consequently, both routes yield limited improvements in perovskite film quality. In contrast, treating intermediate‐phase films during thermal annealing enables both increased grain size and surface defect passivation. We hypothesize that during recrystallization, thiol molecules compete with solvent molecules for coordination with Pb^2+^ species, thereby reducing the chemical activity of free Pb^2+^ in the precursor solution. The resulting decrease in supersaturation suppresses nucleation and promotes crystal growth [16]. Figure 1b shows schematically the as‐deposited wet perovskite film after gas‐quenching and subsequent solid‐state perovskite with thiol vapor annealing. The thiol molecules have the potential to predominantly anchor at the perovskite surface and electronically passivate it [17, 18], as illustrated in Figure 1c. BT vapor introduced in intermediate‐phase yields superior device performance, with average PCEs exceeding 18%, 30 mV open circuit voltage (Voc) increase, and notable improvements in short‐circuit photocurrent (Jsc) and fill factor (FF). Additionally, due to the self‐limiting nature of thiol passivation, no additional anchoring points are available after the initial passivation, preventing the growth of extra layers [17]. Device performance remained relatively constant with exposure times ranging from 3 min to 12 h, Figure S6, thus demonstrating a long processing window that facilitates integration into R2R and offers a fast and scalable strategy highly compatible with continuous R2R processing. While the present results demonstrate the feasibility of integrating thiol vapor treatment into a pilot‐scale R2R process under controlled conditions, vapor‐flow uniformity across wider webs and at higher line speeds has not yet been systematically evaluated. Therefore, maintaining uniform vapor distribution remains a consideration for industrial implementation. Figure 1d illustrates a schematic of a perovskite crystal with thiol molecules anchored at the surface, alongside the final R2R‐printed perovskite film with the HTL on top.

**FIGURE 2:**
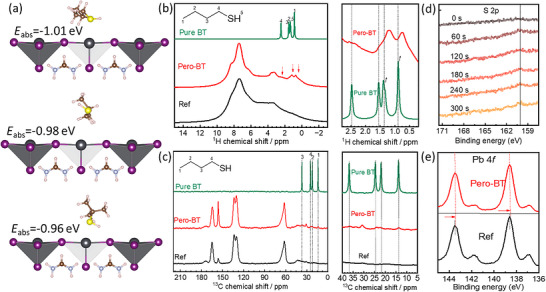
(a) DFT calculated binding energy of three thiols molecules on perovskite surface. (b) ^1^H MAS NMR spectra at 30 kHz (or 0 kHz for the liquid) of: pure BT, Pero‐BT and film without BT treatment and zoom‐in. (c) ^13^C DE NMR spectra at 0 kHz of pure BT, and ^13^C CP MAS NMR spectra at 25 kHz of a Pero‐BT and film without BT treatment. Line broadening: 100 Hz. (d) Depth XPS profile of S 2p with increased etching time in perovskite film. (e) The Pb 4f XPS spectra of perovskite film with and without BT treatments.

The underlying mechanism of how thiol molecules bind to perovskite was investigated using density functional theory (DFT) calculations and spectroscopic analyses. DFT shows that linear BT binds most strongly to the perovskite surface with an adsorption energy of −1.01 eV, versus −0.98 eV for 2M1PT and −0.96 eV for 2M2PT (Figure 2a). Owing to its linear structure and stronger binding affinity, BT is likely to form a more compact and densely packed layer at the perovskite interface through S–Pb coordination, compared to the other two alkylthiols [19]. This explains why BT treatment leads to more effective improvements in film quality compared to its structural isomers. Solid‐state nuclear magnetic resonance (NMR) spectroscopy under Magic Angle Spinning (MAS) was then employed to prove the presence of butanethiol in the Pero‐BT sample and to probe its interaction with the perovskite phase. Compared with solution‐state NMR, solid‐state spectra are generally broader due to chemical shift anisotropy and dipolar coupling between nuclei, leading to overlapping resonances in ^1^H MASNMR spectra. Nevertheless, distinct proton resonances corresponding to the film components could be assigned. In the reference sample, Figure S7, characteristic signals of the PET substrate appeared at 8.3 and 3.4 ppm, while formamidinium (FA) and methylammonium (MA) cations exhibited resonances at 7.4–8.3 ppm and 6.2–3.4 ppm, respectively [20, 21]. For the BT‐treated film, four additional broad resonances at 2.3, 1.5, 1.2, and 0.7 ppm are observed, Figure 2b, corresponding to the alkyl protons of BT. Their broadening indicates immobilization on the surface. All BT resonances are slightly shifted upfield, particularly those of the terminal –CH_2_–SH group (by 0.2 ppm), which may be caused by coordination of the thiol to Pb^2+^ cations. Interestingly, ^1^H MAS NMR revealed significantly longer longitudinal relaxation times (T_1_) for FA protons in BT‐treated films (16.4 ± 1.1 s) compared to untreated ones (5.2 ± 0.5 s), indicating enhanced crystallinity. The ^13^C CP MAS spectra, Figure 2c, were then explored to confirm the presence of BT. In addition to PET (164.6, 133.6, 130.2, 61.8 ppm) [22], FA (155.8 ppm) [23], and MA (30.5 ppm) signals [24], four new resonances appeared at 36.6, 24.6, 21.9, and 13.8 ppm are detected, corresponding to BT carbons. A slight upfield shift (∼0.6 ppm) of the C(4) carbon adjacent to –SH again suggests surface binding. Collectively, these results support the thiol‐assisted vapor annealing mechanism, where thiol molecules dynamically interact with the wet perovskite film to regulate crystallization and improve structural order, and passivate undercoordinated Pb sites.

Moreover, depth‐profiling x‐ray photoelectron spectroscopy (XPS), Figure 2d, was applied to explore the spatial distribution of sulfur in the final films. Sulfur signals were consistently detected from the surface down to 300 s of etching, indicating that thiol‐derived species are present beyond the surface and extend into the film. Table S2 summarizes the quantitative sulfur concentration in the perovskite film derived from XPS depth profiling, indicating that sulfur is present at the surface and persists throughout the whole thickness. Since sulfur incorporation in the bulk can only occur along grain boundaries, this observation provides strong evidence that thiol molecules penetrate grain boundary regions during the annealing process. We further observed a slight peak shift from 138.7 and 143.6 eV to 138.5 and 143.4 eV toward the lower binding energy of the Pb 4f orbital, Figure 2e, resulting from the electron transfer between the sulfur atom and the uncoordinated Pb^2+^, indicating the interaction between Pb and S [25]. In addition, a lower concentration of metallic Pb^0^ on the surface of the VP‐GBA treated perovskite film was observed compared to the pristine one, indicating relatively effective suppression of PbI_2_‐to‐Pb° conversion and shield‐protection against beam‐induced degradation under XPS measurements [26]. It is visualized by energy dispersive spectroscopy that sulfur is found homogenously covering all the grains, preferentially interacting with defect‐rich regions such as grain boundaries, which reinforces the intergranular toughness against tensile stress, Figure S8.

Mechanical resilience and flexibility are critical for perovskite films in R2R fabrication. Typically, Young's modulus and hardness are employed to quantify the flexibility figure of merit of thin films. A lower Young´s modulus leads to a longer yield strain ε_*y*_ according to the ratio of σ_*y*_/*E*, under comparable yield strengths σ_*y*_, indicating that the material exhibits greater elastic resilience. Yield strength σ_*y*_, is the maximum stress a material can endure before breaking or deforming permanently [27]. It is typically measured through tensile tests but can also be estimated using an empirical formula commonly applied to ceramics: [28] $\sigma_{y}=\frac{1}{2}\;\left(1-2\upsilon \right)\cdot P_{m}$, where υ is Poisson´s ratio of perovskite [29] and *P_m_* is the mean contact pressure. During the plastic stage, the material deforms nonlinearly, with the ultimate strength representing the maximum stress the material can withstand. Beyond this point, necking occurs, leading to fracture. According to the Lawn‐Evans‐Marshall theory [30], fracture toughness $K_{IC}=\alpha \cdot \sqrt{\frac{E}{H}}\cdot \frac{P}{c^{3/2}}$ is introduced to quantify a film´s ability to resist crack initiation and propagation under applied stress. Here, α is an empirical constant (∼0.016 from finite element simulations), *P* is the applied force, and *c* is the crack length created after indentation. A higher *K*_IC_ implies that the film can absorb more energy during deformation, thereby enhancing its bending durability under mechanical stress.

**FIGURE 3:**
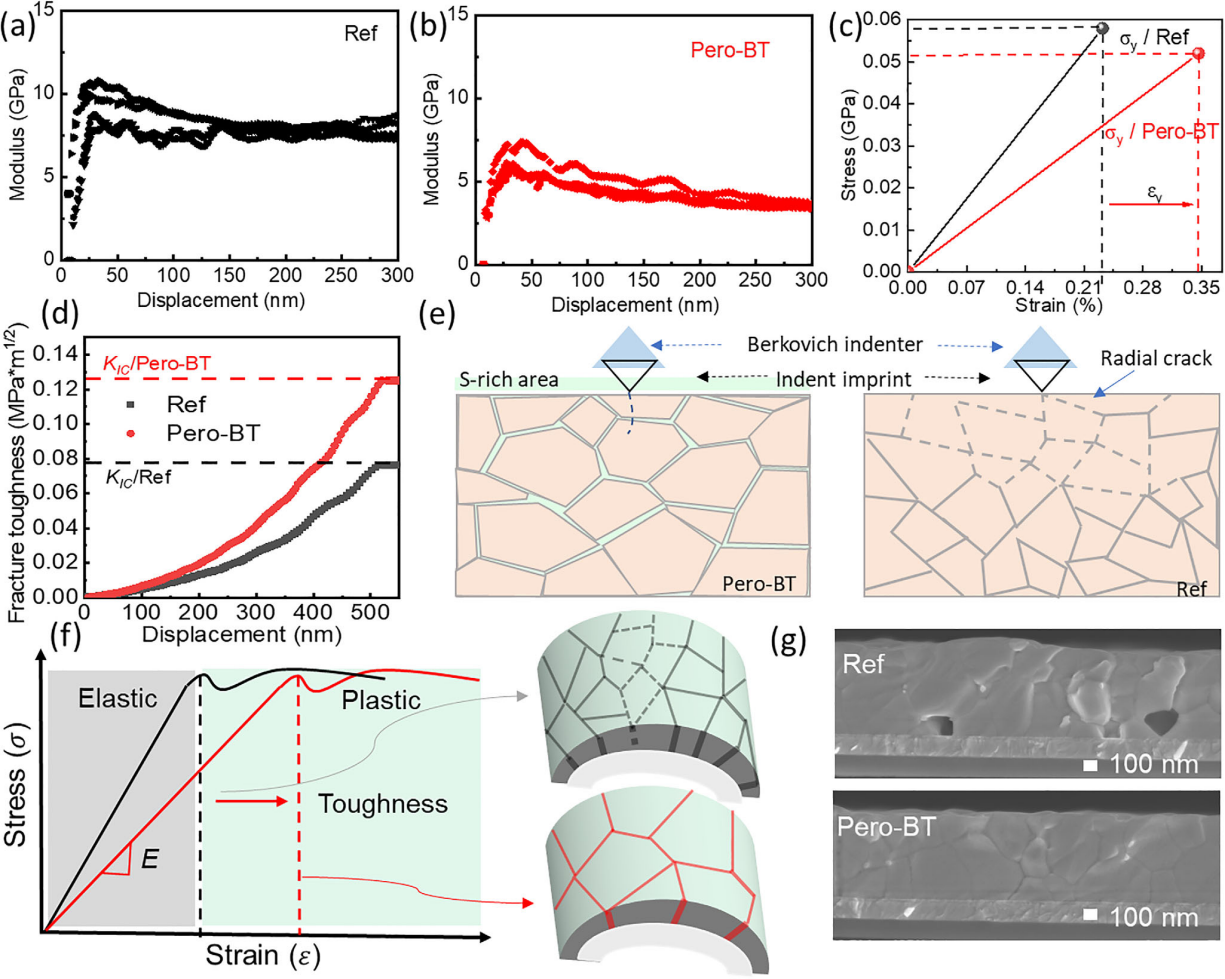
(a) Young's modulus of perovskite film with and without (b) BT treatment measured by nanoindentation. (c) Stress versus yield strain plots for the VP‐GBA‐treated and pristine perovskite films. (d) Fracture toughness of perovskite film wi/wo BT vapor. (e) Schematics of crack formation in perovskite film under indentation. (f) The schematic of stress versus strain plot and effects of grain boundaries on film crack formation under curvature. (g) Cross‐sectional SEM of perovskite wi/wo BT vapor.

To quantify the mechanical properties, we performed Nanoindentation measurements on three different areas on each perovskite sample (1 cm × 1 cm) to assess homogeneity. In each region, nine indents were made at an indentation depth of 500 nm. The average hardness *H* of the perovskite film with VP‐GBA treatment decreases by 20% from 0.10 to 0.12 GPa, Figure S9. The decrease in Young's modulus *E* by approximately 60% from around 8.8 to 3.5 GPa upon BT vapor treatment is quite significant overall, Figure 3a,b. Statistics of each indentation can be found in Table S3. The corresponding yield strain ε_*y*_ of the VP‐GBA‐treated perovskite film is significantly increased by 50%, compared to the pristine perovskite film, as shown in Figure 3c. Accordingly, the fracture toughness was calculated by combining with the half‐penny crack formation model [10]. The resulting cracks after a 500 nm indentation depth are shown in Figure S10, with an average crack length of approximately 0.89 µm for the VP‐GBA‐treated perovskite, in contrast to 1.47 µm for the reference sample. Figure 3d presents the calculated fracture toughness, showing an improvement from approximately 0.08 to 0.13 MPa×m^1/2^ after BT treatment. The enhanced elastic resilience and structural toughness enable the perovskite film to withstand strong mechanical stress under indentation, as illustrated in Figure 3e, validating its eventual processing through R2R methods. This ensures improved mechanical robustness while maintaining functional integrity in C‐fPSM.

Figure 3f schematically illustrates the stress–strain behavior of perovskite films with different Young's moduli, corresponding to variations in grain size under bending deformation. According to Hall‐Petch theory, which is often applied in crystalline metal materials, an increase in grain size results in a ´´softening´´ phenomenon [31, 32]. This approach is based on the principle that grain boundaries serve as barriers to dislocation motion. The accumulation of dislocations within a grain alters the local stress distribution, which subsequently activates dislocation sources in neighboring grains and deforms the intergranular region. By tailoring the grain size, the accumulation of dislocations at grain boundaries can be effectively controlled. We observed a similar trend in our perovskite films. The cross‐sectional SEM images of the perovskite are shown in Figure 3g and the grain size distribution statistics are shown in Figure S11. The BT‐treated perovskite shows a more compact inter‐grain stack with an increased average grain size by approximately 100 nm compared to the pristine perovskite. This observation supports a thiol‐assisted vapor‐annealing mechanism in which BT infiltrates grain boundaries and forms S–Pb coordination, thereby regulating crystallization across the entire film (including the bulk) and passivating under‐coordinated/metallic‐forming Pb defects. Collectively, these effects lead to enhanced structural order and improved mechanical robustness. Accordingly, enlarging grain size is an effective route to improve the flexibility and toughness of perovskite films by lowering stress concentration and reducing crack initiation.

The critical role of BT‐based VP‐GBA treatment in tailoring the crystallographic morphology of the perovskite thin film and the crystallinity of perovskite was further measured by x‐ray diffraction, Figure S12, and shows an approximate two‐fold increase in diffraction intensity for all diffraction peaks. The PbI_2_ phase is notably suppressed, consistent with the SEM images. A close inspection of the (100) diffractive peak shows a significant reduction of full width at half maximum (FWHM) from 0.22° to 0.19°, which indicates the improved crystal size and quality after VP‐GBA treatment.

The trap density of perovskite is estimated by space charge limited current (SCLC) with the equation $N_{t}=2\varepsilon_{0}\varepsilon_{p}V_{\mathrm{TFL}}/eL_{P}^{2}$, where ε_0 _is the vacuum permittivity, *e* is the electron charge, ε_*p*_ is the relative permittivity of perovskite, and *L_P_* is the perovskite layer thickness. The trap‐filling limited voltage (*V*_TFL_) can be quantified from dark I‐V curves by using the electron‐only device stack of PET/IMI/SnO_2_/Perovskite/PCBM/Carbon under a relatively stabilized regime without ion migration interference. Figure S13 displays a logarithmic presentation of the dark I‐V curves, contrasting devices with and without VP‐GBA treatment of the perovskite film. The estimated electron‐type trap density in perovskite decreases from 7.7×10^15^ cm^−3^ for the pristine perovskite to 3.1×10^15^ cm^−3^ for Pero‐BT. This is consistent with the enhanced polycrystalline perovskite morphology, which features fewer grain boundaries and, consequently, fewer trap states typically associated with these boundaries.

**FIGURE 4:**
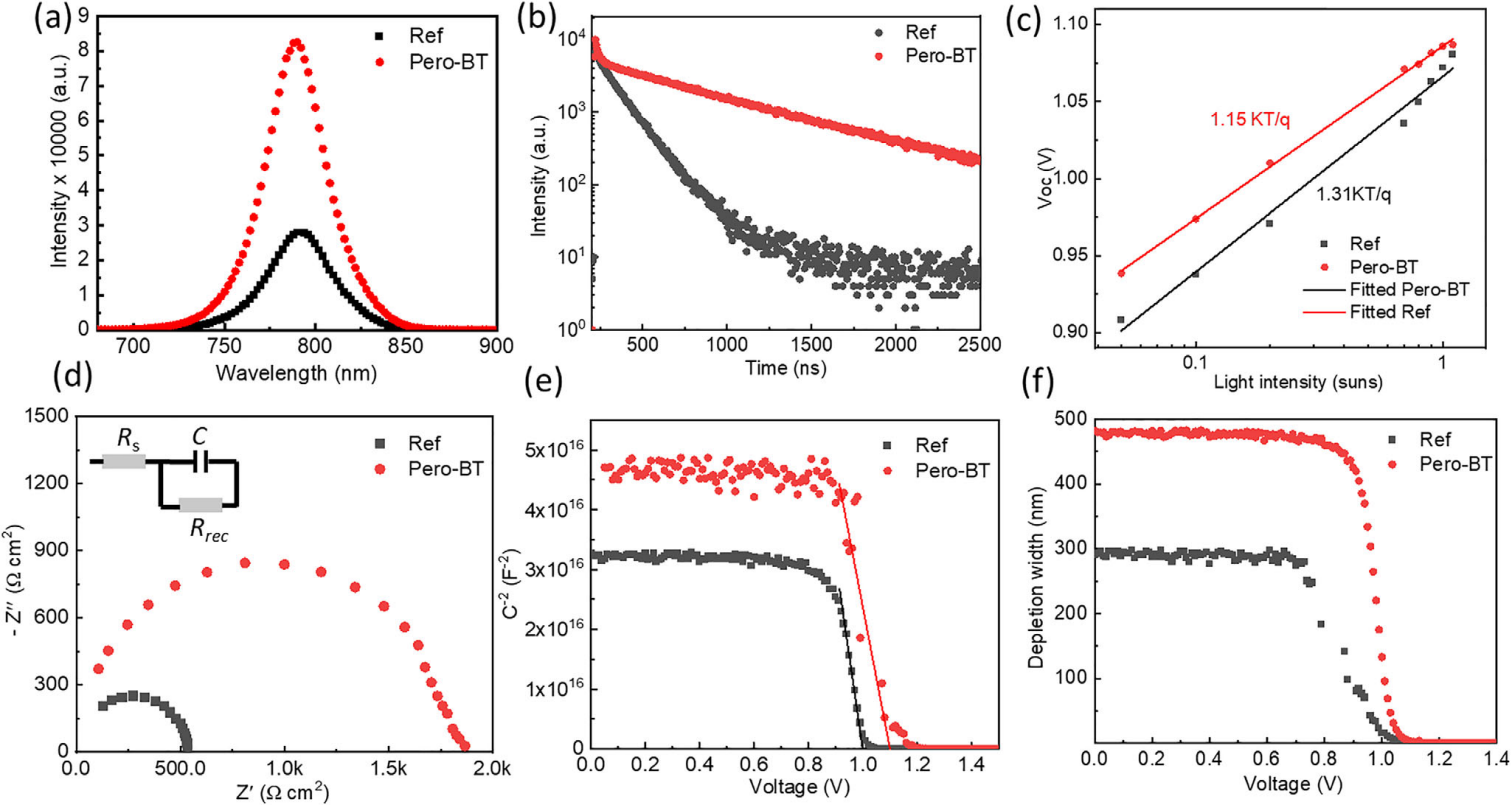
(a) Steady‐state PL spectra of perovskite wi/wo VP‐GBA treatment. (b) Time‐resolved PL of perovskite wi/wo VP‐GBA treatment. (c) Light‐intensity dependence of the Voc measured by I‐V scan under increased light intensity from 0.01 sun to 1 sun. (d) Nyquist plots of perovskite solar cells prepared on bare perovskite and perovskite with BT vapor. The inset shows the corresponding equivalent circuit. (e) Mott–Schottky curves and (f) depletion width of perovskite ‐based devices w/wo VP‐GBA treatment.

With the improved perovskite film quality achieved through thiol treatment, we then quantified charge carrier recombination by combining steady‐state and transient photoluminescence (PL) measurements. As shown in Figure 4a, the VP‐GBA‐treated perovskite film exhibits an approximately fourfold increase in steady‐state PL emission intensity compared to the reference film, while Figure 4b shows a significantly enhanced charge carrier lifetime. We further studied the carrier lifetime under low excitation laser intensity (5.3 nJ cm^−2^), Figure S14. The results show further enhanced lifetime in second‐order recombination regime for VP‐GBA‐treated perovskite, indicating significant suppression of charge trapping. However, in the case of pristine perovskite film, there is barely any improvement in the lifetime, which is most likely due to severe carrier trapping effects, resulting in an insensitivity to varying excitation intensity. When crystal size increases and the surface is passivated, the relative contribution of surface recombination decreases, and the recombination pathway shifts more toward the bulk, which shifts recombination more towards higher‐order (bimolecular) recombination processes and the decay time becomes intensity‐dependent, which can further improve device performance. The suppressed non‐radiative recombination also resulted in a reduced diode ideality factor (*n*_id_), as shown in Figure 4c. The *n*_id_ for the VP‐GBA‐treated flexible device was 1.15, while that for the untreated device was 1.31. It is suggested that recombination losses are minimized due to improved diode quality, which directly translates into superior solar‐cell performance.

To gain deeper insight into carrier recombination characteristics in solar cells, electrochemical impedance spectroscopy (EIS) was employed to investigate their electrical properties. The Nyquist plots, Figure 4d, are obtained by sweeping the frequency from 0.1 Hz to 1 MHz at open circuit conditions in the dark. The data are interpreted using an equivalent circuit model, which assigns the semicircle to the perovskite interface, represented by a recombination resistance (*R*_rec_) and a capacitance [33]. The recombination resistance increases from 297.3 Ω cm^2^ for the unmodified perovskite to 956.9 Ω cm^2^ for the VP‐GBA‐treated perovskite. This indicates that the VP‐GBA‐treated perovskite effectively inhibited undesired charge recombination loss, contributing to the enhanced open Voc.

As indicated by the Mott‐Schottky plot, Figure 4e, VP‐GBA‐treated solar cells show higher built‐in potential (*V*_bi_) than the unmodified devices, which are increased from 1.0 to 1.08 V for the Ref and Pero‐BT device. The trap density is reduced by a factor of 1.62, estimated using the equation of $N_{t}=\frac{2}{e\varepsilon_{0}\varepsilon_{p}}\;{\left(\frac{dC^{-2}}{dV}\right)}^{-1}$, where *C* is measured capacitance per unit area and *V* is applied voltage. The difference arises because SCLC and Mott–Schottky measurements probe different defect populations: SCLC mainly reflects bulk deep traps, while Mott–Schottky analysis is more sensitive to interfacial or shallow states. Nevertheless, both methods consistently indicate a significant reduction in trap density after BT vapor treatment. The calculated *W*_d_ in the device as a function of the applied voltage is plotted in Figure 4f, which is obtained by capacitance‐voltage (C_p_‐V) analysis [34, 35] (details in Supporting Information‐Note). Under the short‐circuit condition, the *W*_d_ is determined to be 480 nm for the VP‐GBA‐treated devices, while that of the reference device is 295 nm. Under maximum power point, the *W*_d_ of the pristine device decreases to 42.8 nm, while that of the VP‐GBA‐treated device is 128.1 nm. These results highlight that the increased Voc in VP‐GBA‐treated solar cells may be attributed to the increased depletion width in the bulk perovskite, improving charge extraction.

Through the above discussions, it is evident that thiol treatment promotes uniform grain growth and smooth grain boundary transitions by reducing grain boundary energy and repairing surface defects. We further study the contact between the perovskite and the top functional layers. The topological view of the perovskite film is measured by confocal microscopy, Figure S15, where the roughness slightly decreases from 18.3 to 17.2 nm after VP‐GBA treatment. The subsequent deposition of HTL onto VP‐GBA‐treated perovskite demonstrates relatively uniform morphology with a narrow height distribution, indicating improved interfacial contact, Figure S16. We further evaluate the adhesion between the BT‐treated perovskite and the HTL using a tape test. First, 100 grid patterns were created on the perovskite film using a femtosecond laser, an adhesive tape was then pressed onto the patterned film to ensure firm contact and subsequently peeled off quickly at a 90° angle. Due to the strong adhesion between the adjacent layers, no HTL material was removed from the BT‐treated sample, as shown in Figure S17. Microscopically, FTIR spectra of PEDOT layers deposited on thiol‐treated perovskite films display subtle yet consistent spectral changes at 1227 and 720 cm^−1^, corresponding to C‐S stretching and S‐C out‐of‐plane deformation modes, respectively, as shown in Figure S18. These features suggest possible chemical interactions between the alkyl‐thiol species and PEDOT. We propose that the alkyl chains of thiols may interpenetrate or form non‐covalent interactions (e.g., van der Waals forces) with the PEDOT matrix, enhancing interfacial adhesion [36].

**FIGURE 5:**
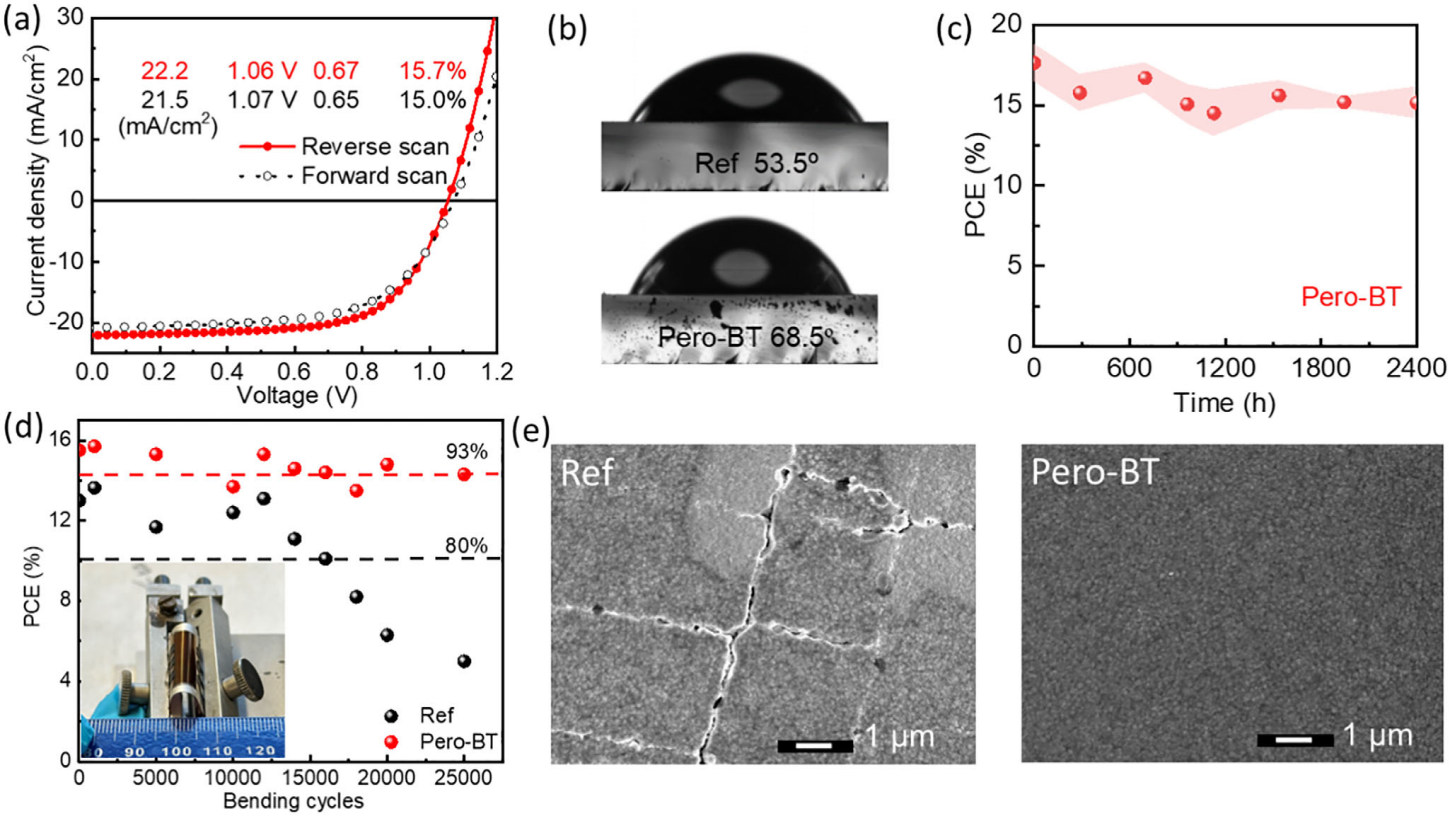
(a) Champion current‐voltage (I‐V) curves of a R2R printed solar cell. (b) Water contact angle of perovskite film with/without VP‐GBA treatment. (c) Shelf‐lifetime of VP‐GBA‐treated perovskite solar cells without encapsulation. (d) PCE of flexible solar cells as a function of mechanical bending cycles. (e) SEM images of the perovskite film with HTL after the 25000 bending tests.

Finally, we turn our attention to demonstrating the PV performance and mechanical stability of the carbon‐based flexible device. We have previously compared metal and carbon electrodes (Figure S19) and found that, although Ag‐based devices show slightly higher Jsc, carbon‐electrode devices achieve comparable or slightly better PCE due to improved FF. For detailed comparisons on efficiency, environmental stability, and mechanical durability, please refer to our earlier work [12, 13]. As shown in Figure 5a, the device performance of the R2R printed solar cells with carbon electrode, shows a record PCE of 15.7%, Voc of 1.06 V, FF of 0.67 and Jsc of 22.1 mA/cm^2^, compared to a reference PCE of 12.0% along with Voc of 1.01 V, FF of 0.54 and Jsc of 21.9 mA/cm^2^ (Figure S20). The external quantum efficiency spectrum and integrated Jsc for the champion device are plotted in Figure S21. The unit cell with BT vapor treatment shows a stable steady‐state power output through 350 min of photocurrent measurement at the maximum power point, Figure S22.

As shown in Figure 5b, the VP‐GBA strategy makes the perovskite surface more hydrophobic, as indicated by the increase in the water contact angle from 53.5° to 68.5°. The increased hydrophobicity is due to the thiol groups' strong preference for bonding with the uncoordinated Pb^2+^ ions at the perovskite surface, leaving the four‐carbon chains oriented upwards, which leads to a hydrophobic surface that can effectively block moisture ingress into the device and improve adhesion with HTL. As shown in Figure 5c, the BT vapor‐treated device without encapsulation exhibits superior environmental stability under ambient air (20%–30% RH, ∼25°C), showing only ∼7% efficiency loss after 2400 h of storage, whereas the reference device degrades much faster (Figure S23). Furthermore, the light stability was evaluated under continuous 1 sun illumination following the ISOS‐L‐1 protocol. The treated device retains 96% of its initial PCE after 1000 h of operation, while the reference devices retain 80% (Figure S24).

Most importantly, VP‐GBA‐treated flexible devices show superior mechanical stability due to improved fracture toughness as discussed above, maintaining 93% of their initial PCE even after 25 000 bending cycles, at a radius of 5 mm, whereas only 80% was left after around 15000 bending cycles for the control device, Figure 5d. To contextualize the mechanical durability of our VP‐GBA treated flexible devices, we summarized recent reports of bending tests on flexible perovskite devices (Table S4). Among these, most literature reports test at a bending radius of 5 mm with a maximum of ∼10 000 cycles, whereas our devices demonstrate robust mechanical stability up to 25 000 cycles while retaining a high fraction of PCE. This is the record among all mechanical stability reports for flexible perovskite devices to date. As shown in Figure 5e, the morphology of VP‐GBA‐treated devices does not show any signs of cracks or fracture even after long‐term severe mechanical compression and tension, whereas the device without VP‐GBA treatment shows clear signs of cracking.

Ensuring uniformity in the perovskite film over large‐area substrates is critical for the fabrication of high‐performance modules, as all subcells in a series‐connected architecture must operate consistently to minimize resistive and mismatch losses. To evaluate the spatial uniformity of the perovskite layer, hyperspectral PL imaging was performed on a 5 cm × 5 cm substrate along the coating direction‐tracking from the coating start point to the end of the coating area, as shown in Figure S25. Specifically, the VP‐GBA‐treated perovskite film exhibits a more uniform PL response across its entire length, with consistent PL peak positions from the start to the end edge, as shown in Figure S26. In contrast, the untreated perovskite film shows a gradual decrease in PL intensity along the scan direction, accompanied by noticeable shifts in the PL peak position, indicating significant spatial inhomogeneity in both optical emission and film quality.

**FIGURE 6:**
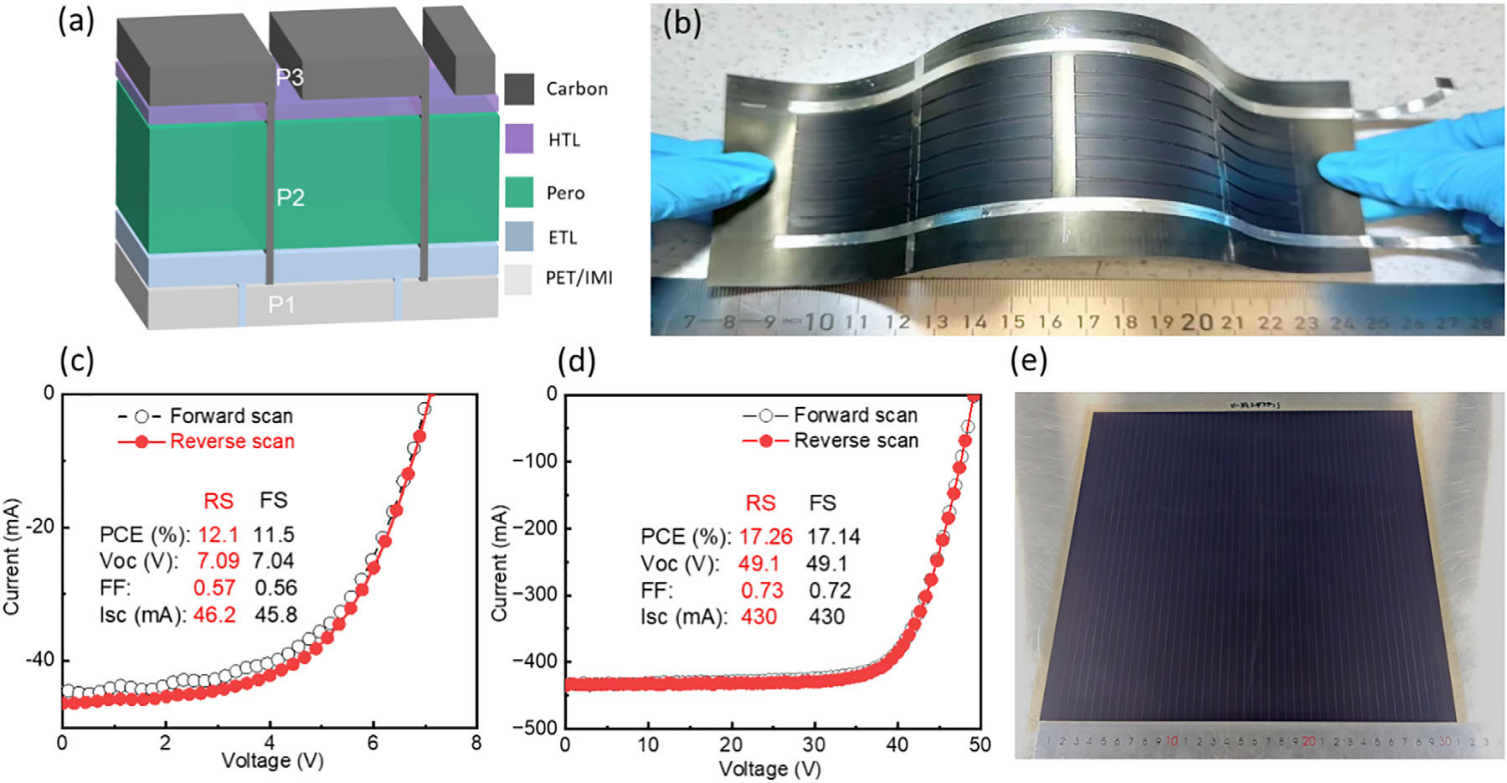
(a) Cross‐sectional schematic drawing showing how individual cells are connected in series to form thin‐film solar modules. (b) A photograph of electrically connected C‐fPSM with a total area of 100 cm^2^ in n‐i‐p layer stacks. (c) I‐V curves of the champion flexible module based on n‐i‐p structure are measured with forward scan and reverse scan. (d) J–V curves of the flexible module with the p‐i‐n device stack on a 900 cm^2^ large‐area. (e) Photograph of the 900 cm^2^ flexible module with the p‐i‐n structure.

In solar modules, the individual solar cells are connected in series, which is achieved by separating the electrodes and functional layers at various positions. In Figure 6a, the schematic diagram illustrates the interconnection of the C‐fPSM, which displays the configuration of the P1, P2, and P3 lines required for the electrical interconnection of adjacent cells. The geometric fill factor (GFF) is introduced for modules as a critical indicator of the overall module efficiency, and represents the ratio of a solar module´s active (i.e., power‐generating) area to its total aperture area. The PET/IMI substrate is first patterned by laser ablation to define the bottom electrodes of the individual cells (P1). ETL is then coated onto the patterned substrate, followed by the application of the perovskite with VP‐GBA treatment and the HTL, using a slot‐die R2R process. Subsequently, femtosecond laser ablation is used to etch the P2 lines that connect the top electrode (carbon) of one cell with the bottom electrode (IMI) of the adjacent cell. Finally, the top carbon electrode is applied via offline stencil printing, using a predefined mask to pattern the P3 lines. Laser patterning of the carbon electrodes after deposition is impractical, primarily due to the strong laser absorption of carbon materials, which induces significant localized heating and poses a risk of damaging the underlying functional layers and the integrity of the P3 scribe. Patterned deposition of the carbon electrodes in‐line with the R2R process will require R2R compatible printing techniques, such as rotary screen printing. Integration of this technique into the R2R printing line is therefore mandatory, as it offers better process compatibility and scalability for carbon electrode deposition. Nevertheless, the stencil printing technology presently used for patterned deposition of carbon electrodes serves to demonstrate the R2R compatibility of our process.

To determine the optimal P2 width, modules with various P2 widths (100, 200, and 500 µm) were tested. The designed minimodule dimensions for the varied P2 width, excluding the effect of GFF, are shown in Figure S27. The positions of P1 and P3 were kept unchanged while P2 width was gradually increased in width. The I‐V curves of each variation are shown in Figure S28, a P2 width of 200 µm showing the highest performance. A narrower P2 (100 µm) might have resulted in higher interconnection resistance due to insufficient contact, which mainly limit FF and ultimately the PCE. As the P2 line is widened to 200 µm, PCE improved due to optimized electrical contact and reduced contact resistance. However, beyond 500 µm, the series resistance no longer improves with further increases in the interconnect area. Hence, we assume 200 µm P2 width creates enough contact area to make a good interconnection. In addition, cell widths of 2, 3, 4, and 5 mm were examined, and it was observed that narrower cells (2 and 3 mm) exhibited higher fill factors than the wider ones (4 and 5 mm) due to reduced lateral resistance. However, when considering the GFF, which increased from 50% (2 mm) and 60% (3 mm) to 67% (4 mm) and 71% (5 mm), the 5 mm cell width ultimately provided the highest overall efficiency due to its relatively high GFF. More details can be found in Figure S29.

With the optimized scalable crystallization and large module dimension design, we achieved a C‐fPSM with a maximum PCE of 12.1%, Voc of 7.09 V, FF of 0.57, and photocurrent of 46.2 mA on an aperture area of 20.25 cm^2^ (effective active area is 15.4 cm^2^), see Figure 6b. The SPO of the champion f‐PSM is illustrated in Figure S30. This represents the highest reported value for printed C‐fPSM to date. Performance statistics of the flexible modules from batch to batch are collected in Figure S31. We also present a 100 cm^2^ flexible module comprising four sub‐modules that are electrically connected in parallel, as shown in Figure 6c. This module achieved a PCE of over 10% as shown in Figure S32. We further validate the VP‐GBA process in inverted flexible modules with the layer stack of PET/ITO/NiO/Perovskite/C_60_/SnO_2_/Cu, achieving a PCE of 17.26% over an active area of 900 cm^2^, as shown in Figure 6d,e. All these investigations point out that the fabrication of flexible modules, with good performance and yield, is viable today. Further improvements along our roadmap project indicate that C‐fPSM have the potential to pass the 20% efficiency milestone. In this context, we emphasize the significance of the VP‐GBA strategy as a key enabling step for achieving high‐yield fabrication of flexible perovskite modules. VP‐GBA treatment effectively suppresses crack formation and enhances toughness during R2R processing by reducing the Young's modulus and increasing elastic resilience, thereby significantly improving mechanical stability. By enlarging grain size, it further improves film morphology and passivates grain boundaries and the top interface, overall boosting the performance and long‐term stability of perovskite solar cells.

## 3 Conclusions

In summary, we successfully demonstrate a scalable, facile, and effective approach for achieving both structural and mechanical stabilization of large‐area C‐fPSM using a pilot‐scale R2R‐intergrated technique. By integrating thiol vapor annealing into the R2R production process, we implement a vapor‐phase grain‐boundary anchoring strategy that suppresses brittle crack initiation and propagation under mechanical stress. This innovative method effectively improves the quality of perovskite film and reduces its Young´s modulus, enhancing its elastic resilience and demonstrating robust fracture toughness, as evidenced by its ability to retain 93% of the initial PCE after 25 000 bending cycles. These improvements result in fully printed C‐fPSM, achieving a record PCE of 12.1% over an aperture area of 20.25 cm^2^ and 10.1% for 100 cm^2^ modules. The approach further demonstrates its universal applicability in inverted architectures, yielding a PCE of 17.26% on a 900 cm^2^ large‐area module. These results highlight the dual benefits of this approach in improving both the photovoltaic performance and the long‐term durability of C‐fPSM. The success of this strategy demonstrates its potential for enabling scalable production of high‐performance and reliable flexible photovoltaic devices for practical applications.

## Author Contributions

Lirong Dong and Viktor Rehm conceived the idea and designed the project. Lirong Dong performed the experiments and wrote the manuscript. Andreas Distler, Hans‐Joachim Egelhaaf, and Christoph J. Brabec supervised the project. Shudi Qiu contributed to SEM measurements. Zexian Han helped with experiments and data processing. Leopold Lahn measured the XPS under Olga Kasian's guidance. Naveen Harindu Hemasiri carried out R2R production together with Michael Wagner. Chaohui Li performed the PL measurements. Nanoindentation was measured by Michael Wurmshuber via Wolfgang Heiss link, both involving mechanical toughness analysis. Cindy‐Ly Tavera‐Méndez carried out and further analyzed NMR results under guidance of Dorothea Wisser. Robin Basu designed minimodule dimension. Sarmad Feroze involved in discussion with module measurements. Fu Yang, Hans‐Joachim Egelhaaf, and Christoph J. Brabec reviewed and revised the manuscript. All co‐authors discussed the results and provided comments on the manuscript.

## Conflicts of Interest

The authors declare no conflict of interest.

## Supporting information




**Supporting File**: Anie72887‐sup‐0001‐SuppMat.docx.

## Data Availability

The data that supports the findings of this study are available in the Supporting Information of this article.
