# Supplementary material for: Vapor‐Phase Grain‐Boundary Anchoring Enables Molecular Toughening and Record Bending Endurance in Pilot‐Scale Roll‐to‐Roll‐Printed Flexible Perovskite Modules
Source: Angew Chem Int Ed Engl. 2026 May 26;65(30):e7292679. doi: 10.1002/anie.7292679 (PMC13383168; doi:10.1002/anie.7292679)
Supplement: Supplementary file 1 — Supporting File: Anie72887‐sup‐0001‐SuppMat.docx. [file ANIE-65-e7292679-s001.docx]

**Supplementary information**

Experimental Methods

**Preparation of perovskite solar cells**

All reagents including material were used without further purification. ITO (Indium Tin Oxide) Coated Glass (Liaoning Yike Precision New Energy Technology Co.,Ltd.) and flexible substrates PET/IMI (purchased from OPVIUS GmbH) were ultrasonically cleaned sequentially for 10 minutes in acetone and isopropanol. Then, the substrates were treated under UV-Ozone box for 10 minutes (flexible substrates for 5mins) to remove organic residues and to enable better wetting. For the standard device an aqueous SnO_2_ nanoparticle solution (Alfa Aesar) was used to prepare the electron transport layer (ETL). The SnO_2_ solution was diluted to 5.0 wt.% and treated in the ultrasonic bath for 10 minutes followed by filtering using a 0.45 µm PTFE filter. The solution was then doctor bladed at 70 °C with 15 mm/s and a gap height of 100 µm. Next, the film was annealed at 150 °C for 30 minutes to form a compact layer. The SnO_2_ on a flexible PET substrate was printed by R2R slot-die. For the R2R production, perovskite is contained equal molar ratio of MAI (Sigma, 98 %) and PbI_2_ (TCI, 99.99%) dissolved in 2-methoxyethanol (2Me, Sigma, 99.8%) with1-methyl-2-pyrrolidinone (NMP, Aldrich, 99.8%) (2Me: NMP, 9:1) to prepare 1.25 M MAPbI_3_ precursor solution and 10% GACl was added as additive. For the perovskite solar cell fabrication, we are using 1.25 M FAPbI3 along with an additional 10% CsCl. The precursor solution was doctor bladed onto the substrate at 4 mm/s and a gap height of 150 µm. The substrate with the still wet film was treated by air blowing for 10 seconds to get a yellowish perovskite intermediate film followed an annealing process at 120 °C for 10 minutes to get the final perovskite film. The perovskite layer on the flexible substrate was subjected to annealing at 100°C for a duration of 10 minutes with BT vapor. PEDOT (poly(3,4-ethylenedioxythiophene)) (HTL solar 3, purchased from Ossila) is being used as the hole transport layer. A gap height of 250 µm and a volume of 30 µl was used for doctor blading. The blade-coating temperature and speeds for HTL were 60 °C with 10 mm/s, following with annealed at 100 °C for 5 minutes. Finally, the carbon paste (Liaoning Yike Precision New Energy Technology Co.,Ltd.) was printed on top of HTL following by annealing on a hot plate at 120 °C for 10 minutes.

**Preparation of flexible perovskite solar modules**

The preparation of flexible perovskite solar modules begins with the patterning of a flexible PET/IMI foil using a femtosecond laser to create P1 lines prior to coating with SnO_2_. Subsequently, the patterned conductive flexible foils undergo cleaning via a CO_2_ laser. The cleaned flexible foil was prewetted by fluoro based-surfactant (Capstone) before coating SnO_2_. The layer of SnO_2_ (3wt%) is then applied onto the clean, patterned PET/IMI using a roll-to-roll slot die at a flow rate of 0.3 ml/min. The resulting SnO_2_ film undergoes transfer to an oven for annealing at 120ºC for a few minutes. Perovskite and HTL are printed by R2R slot-die on SnO_2_ foil afterwards. The stack consisting of PET/IMI/SnO_2_/Pero/HTL is then subjected to patterning of P2 lines using a femtosecond laser power of 850 mW. Finally, carbon electrodes are printed onto the substrate using a stencil mask to create P3 lines, followed by transfer to a hotplate for annealing for 10 minutes at 120 ºC.

**Roll-to-roll fabrication of flexible devices**

Flexible devices were fabricated on a 125 mm wide PET/IMI foil using a R2R process at ambient condition with a controlled relative humidity at 15%. Initially, laser patterning (P1) was performed using a femtosecond laser system (Laser Systems GmbH), with alternating patterning of both cell and module structures. Residual burrs generated during laser scribing were subsequently removed using CO_2_ snow jet cleaning.

A 2.5 wt% colloidal SnO_2_ solution in DI water containing 0.1 V/V% Capstone, was deposited via R2R slot die coating at a roller temperature of 45 °C. The flow rate was set at 0.4 mL/min with a coating speed of 0.5 m/min. The coated substrate was passed to the R2R hot air flow chamber to dry the layer at 120 °C over a 15 mins period. To fabricate the perovskite layer, the SnO_2_-coated PET/IMI substrate was transferred to the R2R coating station. A 0.5 M solution of perovskite in a 2ME and NMP solvent mixture was coated at room temperature, with a flow rate of 0.3 mL/min and a coating speed of 0.4 m/min. Subsequently, the wet film was subjected to compressed air drying using an air knife system operating at 0.4-0.6 bar, followed by thermal annealing in a hot air flow at 100 °C for 10 min, where the 1-butanethiol vapor treatment was conducted. Finally, the HTM layer was deposited at a flow rate of 0.3 mL/min and a coating speed of 0.5 m/min, followed by a final hot air annealing process at 100 °C for 10 min.

**Sheet-to-Sheet Fabrication of Large-Area Modules**

Large-area flexible perovskite solar cells with an active area of 864.0 cm^2^ were fabricated using 30 × 30 cm^2^ PET/ITO substrates. The PET conductive substrates were sequentially cleaned ultrasonically in detergent, acetone, deionized water, and isopropanol for 15 min each, followed by drying with a nitrogen stream.

NiO layer: A metallic Ni target (purity: 99.9%) was used as the source material. The target surface was pre-sputtered for 120 s at 50 W DC power in an Ar atmosphere to remove surface contaminants. Subsequently, a NiO film was deposited onto the ITO substrate by DC sputtering for 500 s at 80 W under a chamber pressure of 3 Pa, with Ar and O_2_ flow rates of 40 sccm and 20 sccm, respectively. The resulting film was then annealed at 100°C for 120 min.

Perovskite layer: The perovskite precursor solution was deposited onto the NiO-coated substrate via meniscus-assisted air-blading at a speed of 8 mm/s, with a 200 µm gap between the blade and the substrate. A 0.5 MPa air-knife was applied to remove excess solvent, followed by annealing at 150°C for 10 min to form the FAPbI_3_ perovskite film.

C₆₀ layer: A 25 nm-thick C_60_ electron transport layer was thermally evaporated at a rate of 0.3 Å/s.

SnO_2_ layer: Tetrakis(dimethylamino)tin (TDMA-Sn) and ultrapure water were used as the tin precursor and oxidant, respectively, in an atomic layer deposition (ALD) process. Each cycle consisted of TDMA-Sn injection/N_2_ purge/H_2_O injection/N_2_ purge (0.4 s / 15 s / 0.2 s / 15 s). The deposition was carried out at 120°C for 145 cycles, followed by annealing at 120°C for 20 min.

Cu electrode: Finally, a 100 nm Cu electrode was deposited by thermal evaporation under a vacuum of 10^-4^ Pa.

**Characterization**

All density functional theory (DFT) calculations were performed using the Vienna Ab initio Simulation Package (VASP) [1,2]. The exchange–correlation potential was treated with the Perdew–Burke–Ernzerhof (PBE) functional within the generalized gradient approximation (GGA) [3]. The interactions between electrons and ions were described using the projector augmented-wave (PAW) method [4]. A plane-wave cutoff energy of 400 eV was applied, and the Brillouin zone was sampled using a 3 × 3 × 1 k-point grid. The energy and force convergence criteria were set to 1×10⁻⁴ eV and 0.02 eV·Å⁻¹, respectively. The DFT-D3 method was employed to account for van der Waals (vdW) interactions [5]. All structural models and charge density distributions were visualized using the VESTA software [6].

The adsorption energy (E_ads_) of the reactive intermediates was calculated using the following equation:

E_ads_ = E_A*_ – E_A_ – E_sub_

where E_A*_ and E_A_ represent the total energies of the system after and before the adsorption of molecule A, respectively, and E_sub_ is the energy of the clean substrate.

Nanoindentation: Nanoindentation was performed using a NanoXP Nanoindenter (Keysight, USA) equipped with a diamond Berkovich tip (Synton MDP, Switzerland) and the continuous stiffness measurement (CSM) option. Indentations were performed to a final indentation depth of 500 nm and the tip shape function has been calibrated beforehand on fused silica. The measured load-displacement data was analyzed after Oliver and Pharr [7]. Average hardness and modulus were taken from an indentation depth of 500 nm to avoid any influence of the substrate. For every cell three different indentation fields with 9 indents each were performed on different areas of the surface to also test for homogeneity. For fracture toughness estimation, additional indents were performed with a diamond cube corner tip (Synton MDP, Switzerland) to an indentation depth of 500 nm.

Solid-state NMR spectra were recorded on an Agilent DD2 500WB spectrometer (11.7 T) with resonance frequencies of 499.9 and 125.7 MHz for 1H and 13C, respectively. A 1H–X–Y MAS probe supporting zirconia MAS rotors with 1.6 mm outer diameter at ambient temperature and 25 to 30 kHz MAS spinning frequency was used. Samples were packed into a suitable 1.6 mm zirconia rotor under inert atmosphere, in a glovebox under nitrogen.

^1^H 1D spectra were recorded at a magic angle-spinning rate (MAS) of 30 kHz for the solid samples and 0 kHz MAS rate (static) for liquid samples. DEPTH sequence for background suppression was used, starting with a π/2 pulse of 2.5 μs followed by two π pulses of 5 μs, pulses were phase cycled according to a combined “EXORCYCLE” and “CYCLOPS” scheme [8]. The number of recorded scans was 16, and recycle delays were set to 5 times the 1H longitudinal relaxation time (T1). Longitudinal relaxation times (T1) of 1H were determined by saturation recovery experiments. The resonance at 0 ppm of sodium 2, 2-dimethyl-2-sila-pentane-5-sulfonate (DSS) was used as chemical shift reference.

^13^C 1D direct excitation (DE) spectra were recorded at a MAS rate of 0 kHz (static) for the 1-butanethiol liquid sample. A π/2 pulse of 2.5 μs, followed by 100 kHz SPINAL-64 decoupling on the 1H channel during the relaxation delay and detection was used. Recycle delays were set to 15 s, and 240 scans were recorded. The resonance at 0 ppm of sodium 2,2-dimethyl-2-sila-pentane-5-sulfonate (DSS) was used as chemical shift reference.

^1^H to ^13^C cross polarization (MAS CP) spectra were recorded at a MAS rate of 25 kHz. Hartmann–Hahn CP matching conditions were optimized on Glycine (1-¹³C, 99%, Aldrich). After a π/2 pulse of 2.5 μs on 1H, the 1H RF was ramped from 77 to 94 kHz, while the RF on ^13^C was kept constant at 68 kHz during a contact time of 3 ms, achieving a Hartmann–Hahn matching condition (n) of +1. 100 kHz spinal-64 decoupling was applied on 1H during acquisition. Recycle delays were set to 1.3 times the 1H longitudinal relaxation time (T1), and between 4100 to 7200 scans were recorded.

XPS measurements were performed (Quantera II, Physical Electronics, Chanhassen, MN, USA) applying a monochromatic Al Kα X-ray source (1486.6 eV) operating at 15 kV and 25 W. The binding energy scale was referenced to the C 1*s* signal at 285.0 eV. Depth Profiling Conditions: Ar, 1kV 1x1, 6 cycles, 1 min/cycle.

trPL spectra measured carried out with a Fluotime 300 system. The samples were excited by the PDL 820 picosecond diode laser with a wavelength of 402 nm at an average incident power of 4 $\mu W$ at a frequency of 20000 kHz.

WF measurements were conducted with a Kelvin probe system SKP5050. The contact potential difference between the tip and the samples was measured. The WF of tip can be confirmed by a gold calibration sample.

PLQY was calculated from an absolute PL measurement with integrating sphere. The films are excited with a 405 nm wavelength laser diode. The intensity of laser was calibrated with a power meter and was adjusted to 80 mW cm^-2^, which is same to the photon flux under AM 1.5G spectra.

EQE spectra were obtained using an EQE measurement system assembled by Enli Technology (Taiwan).

Note 1: Mott-Schottky plot and depletion width calculation

*V*_bi_ is confirmed by Mott-Schottky plot which is 1.0 V and 1.08 V for the ref and target device, respectively. The charge carrier density *N* can be calculated by from the following equation.

$$\frac{1}{C^{2}}=\frac{2(V_{bi}-V)}{A^{2}e\varepsilon\varepsilon_{0}N}$$

The depletion width can be calculated by the equation below:

$$W=\frac{1}{N}\sqrt{\frac{2\varepsilon\varepsilon_{0}(V_{bi}-V)}{q(\frac{1}{N}+\frac{1}{N_{0}})}}$$

Where q is fundamental electron charge, $\varepsilon$ and $\varepsilon_{0}$is the permittivity of free space and perovskite, respectively.


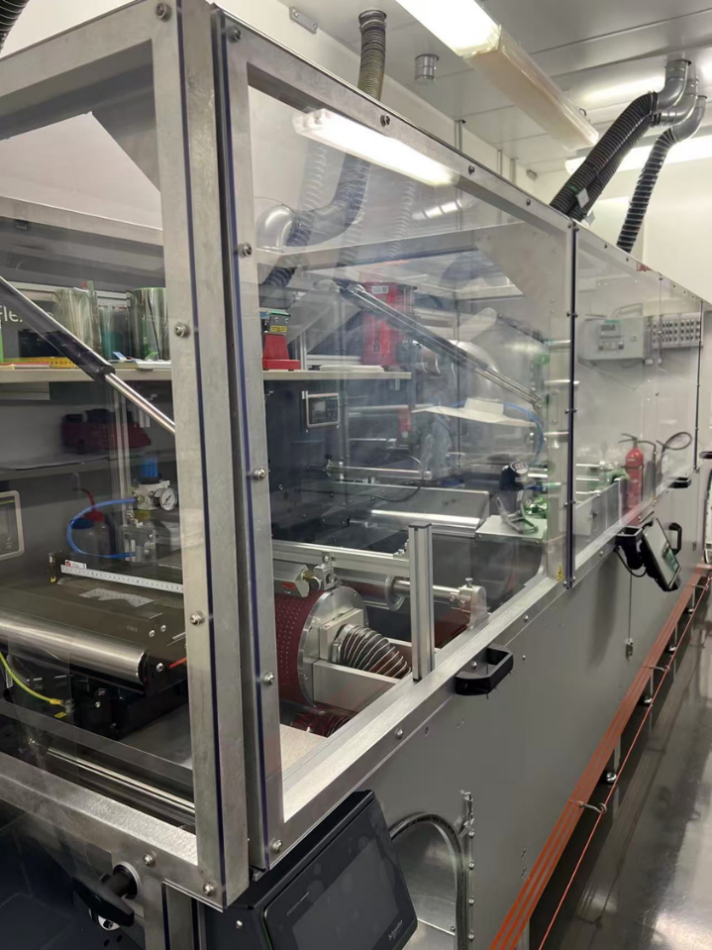


Figure S1. The photograph of the R2R production line with a slot-die and an integrated oven.


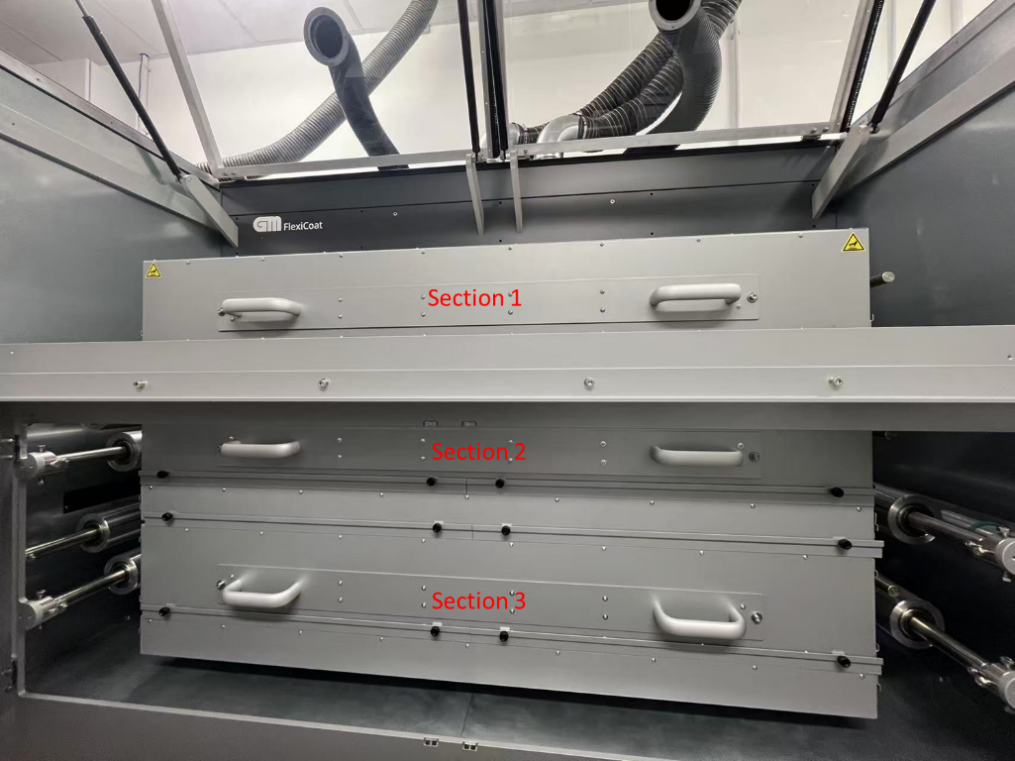


Figure S2. The ovens including three sections on the R2R line are open on both sides so that the foil can pass through and have two exhaust pipes on the top. BT is placed in each section of the oven in an open container for preparing BT vapor.


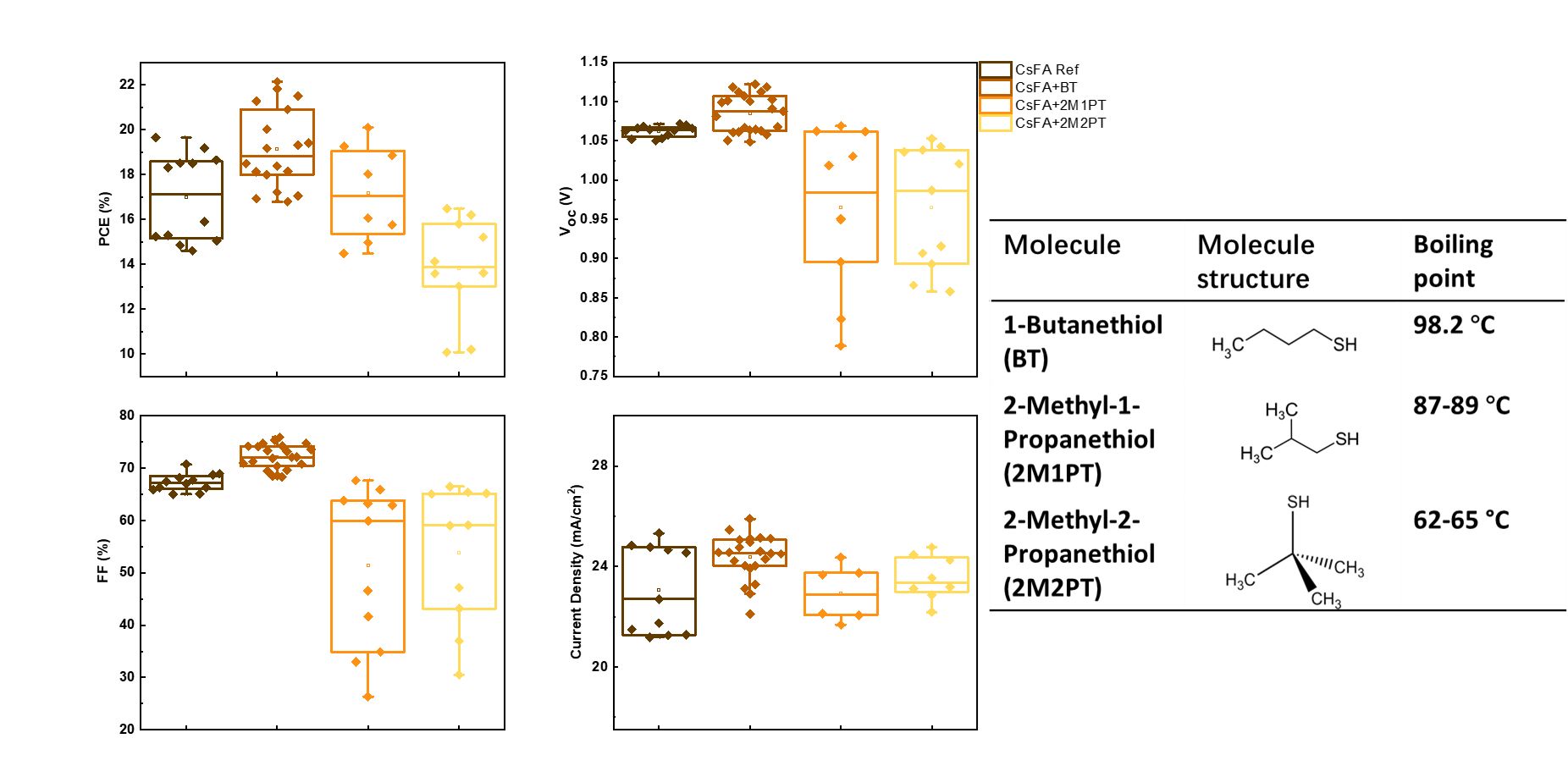


Figure S3. Photovoltaic performance based on three different thiol vapor treated solar cells on rigid substrate and the corresponding thiol molecule structures with their boiling point.


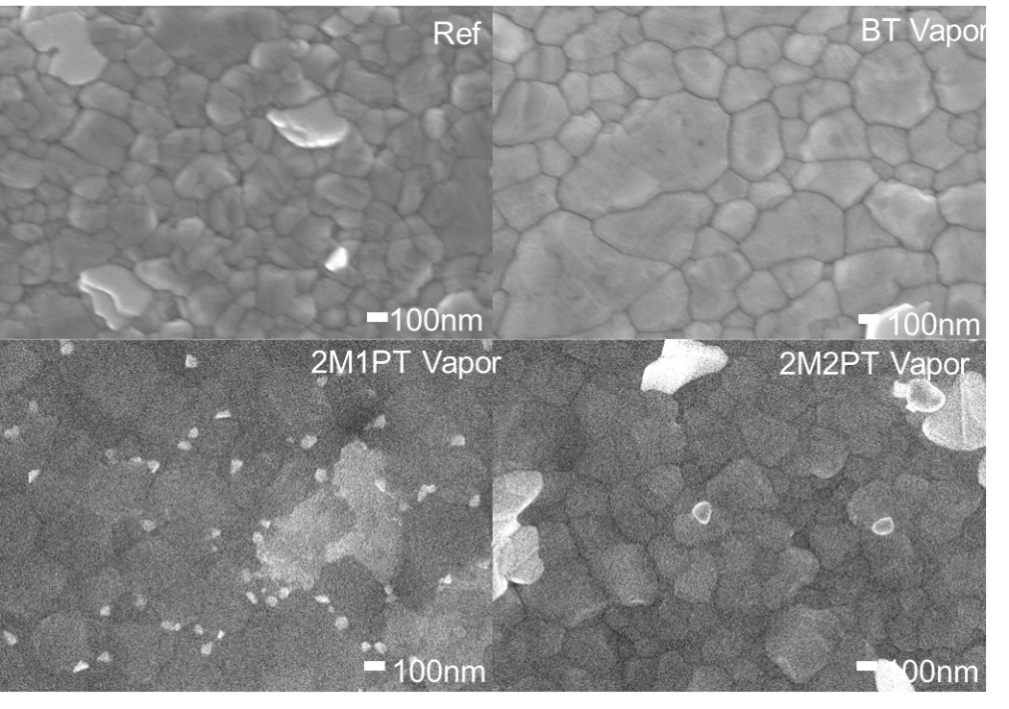


Figure S4. Top-view of perovskite film without (Ref) and with BT, 2M1PT and 2M2PT vapor treatments.


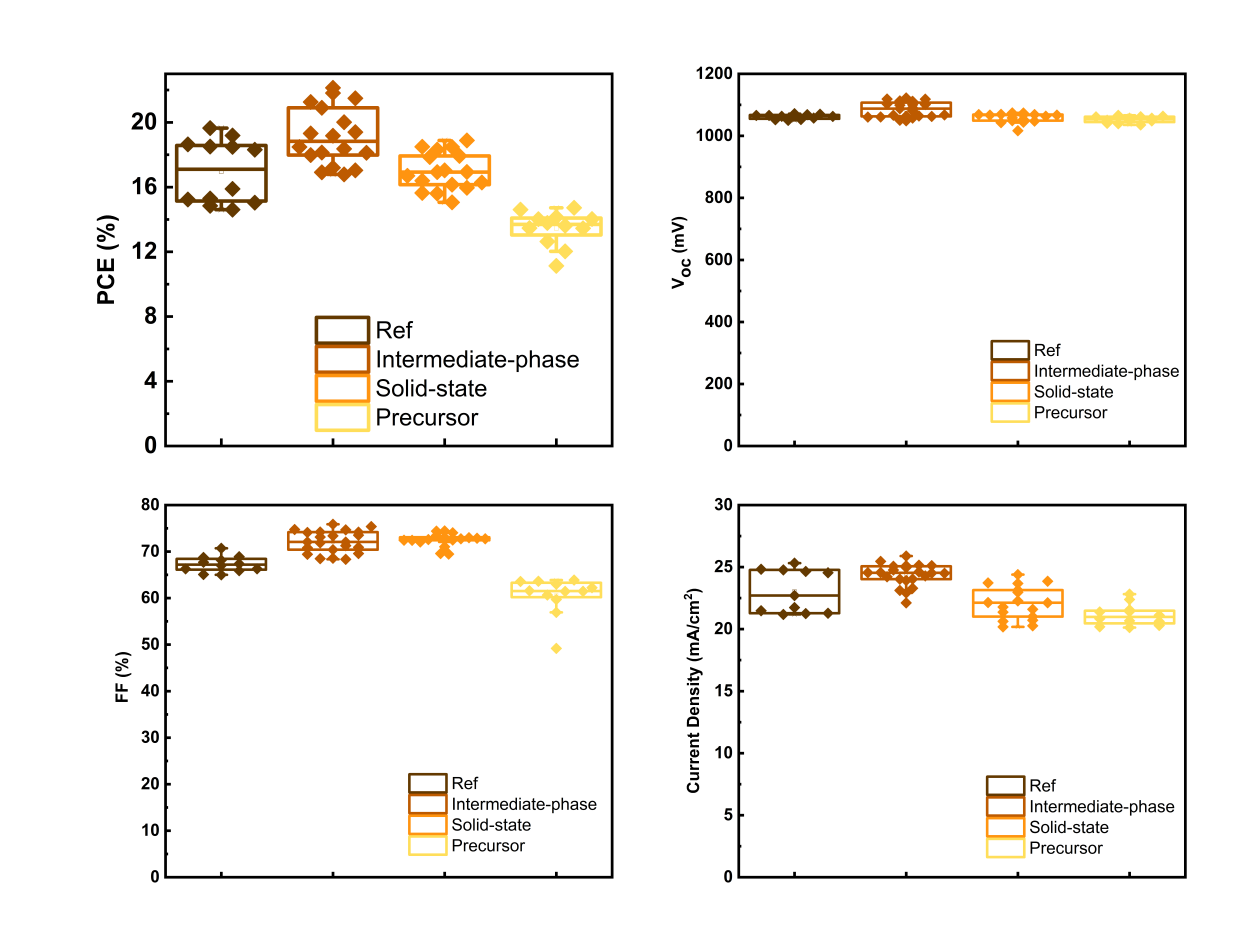


Figure S5. Photovoltaic parameters of perovskite solar cells with BT introduced in different stages of perovskite films.


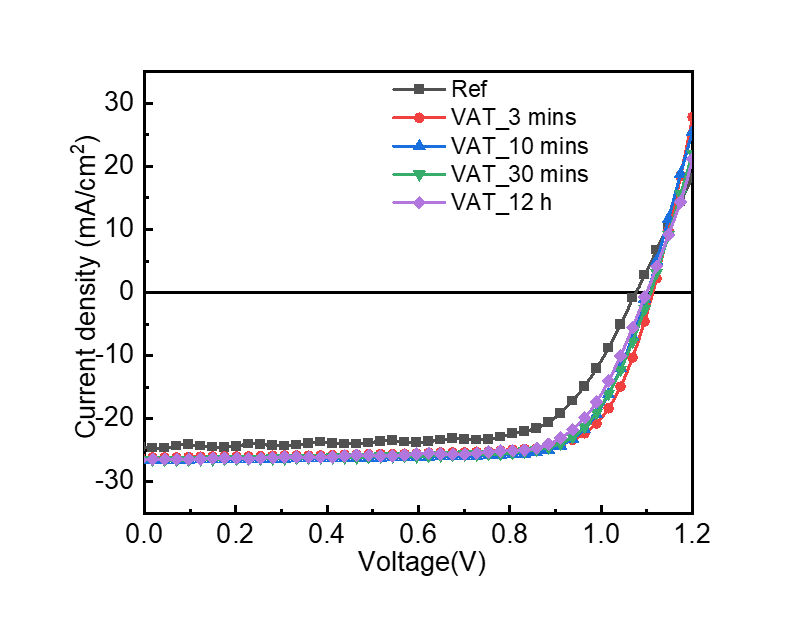


Figure S6. The effects on device performance with increased processing time of VP-GBA treatment on perovskite film from 3 mins up to 12h.


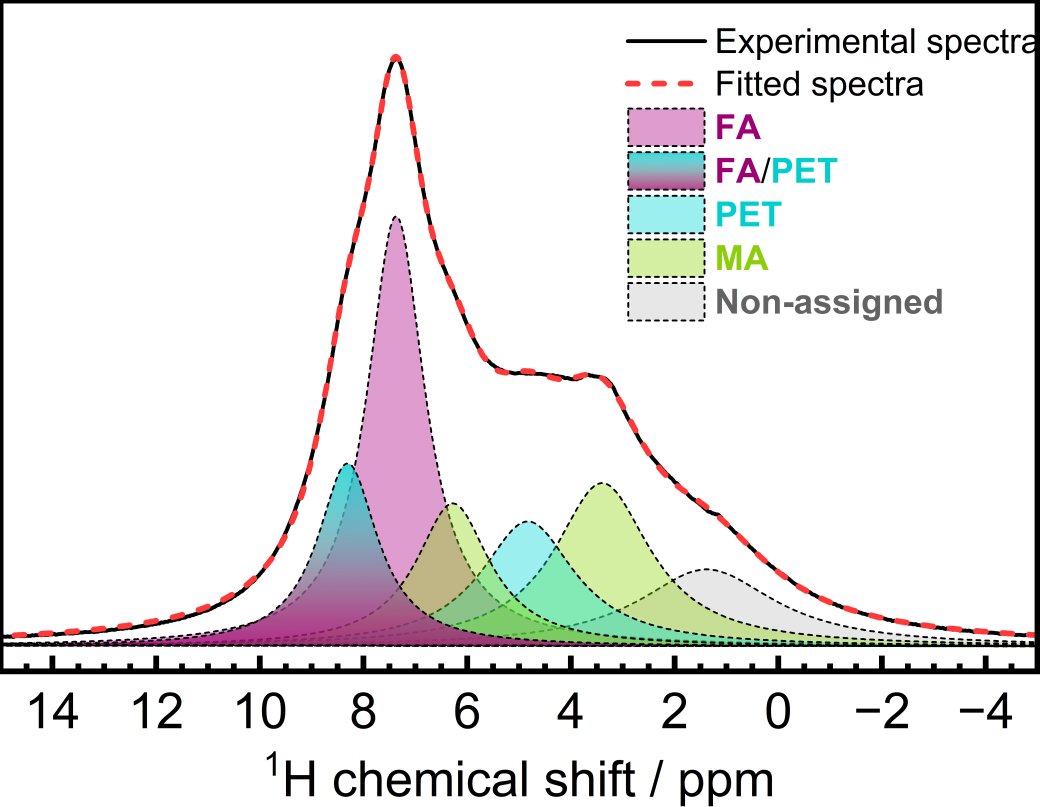


Figure S7. Deconvolution and resonance attribution of the fitted curves for ^1^H ssNMR spectra at 30 kHz MAS of an untreated sample; FA: Formamidinium, PET: Polyethylene terephthalate and MA: methylammonium.

Figure S8. (a) C, I, Pb, N and S elements mapping on perovskite surface and (b) BT vapor treated perovskite film measured by energy dispersive spectroscopy (EDS). (c) The sulfur element distribution was extracted from (b) EDS mapping using ImageJ.


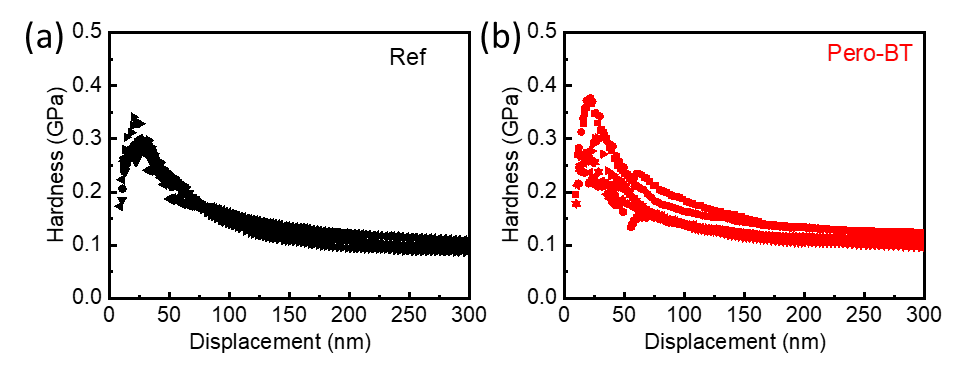


Figure S9. (a) Statistical Hardness of perovskite film (1 cm × 1 cm) and (b) perovskite film with VP-GBA treatment measured by nanoindentation.


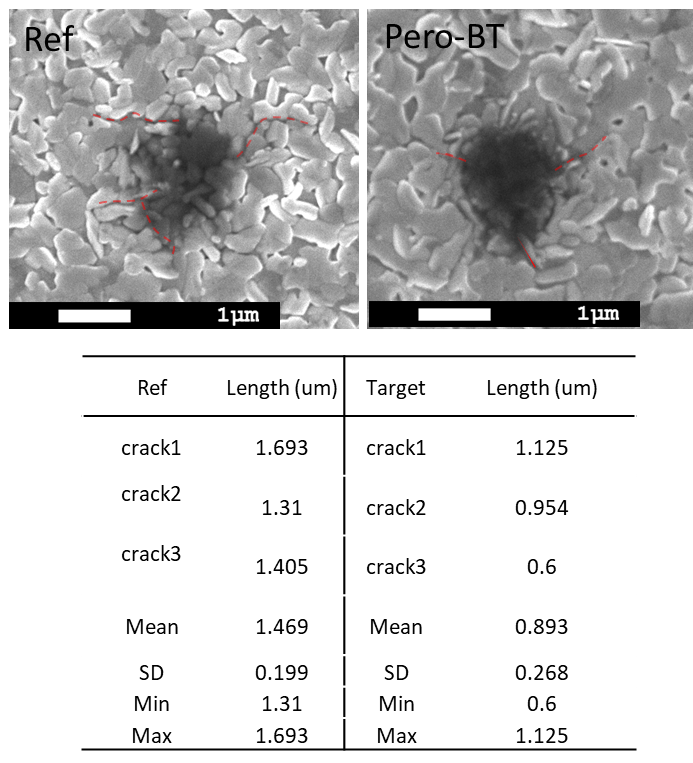


Figure S10. The SEM picture of cracks and extracted crack length formation of perovskite film with and without VP-GBA treatment under indents of 500 nm.


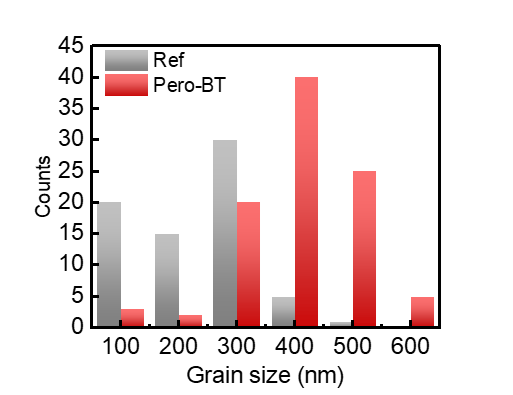


Figure S11. The statistics distribution collection of LHP grain size with and without VP-GBA treatment.

Figure S12. XRD pattern of perovskite and FWHM values on (100) plane diffraction peak of perovskite wi/wo VP-GBA treatment.


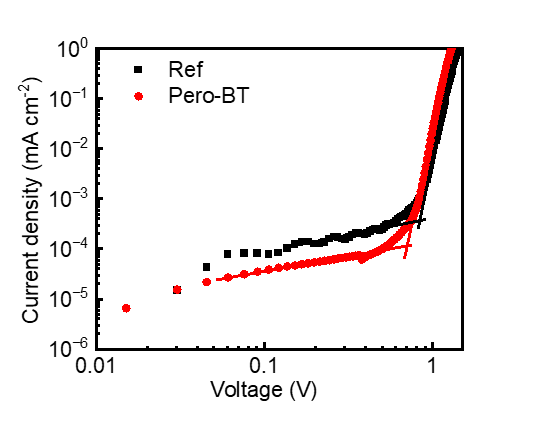


Figure S13. Space charge limited current (SCLC) measurements based on device stack of PET/IMI/SnO2/Perovskite/PCBM/Carbon.


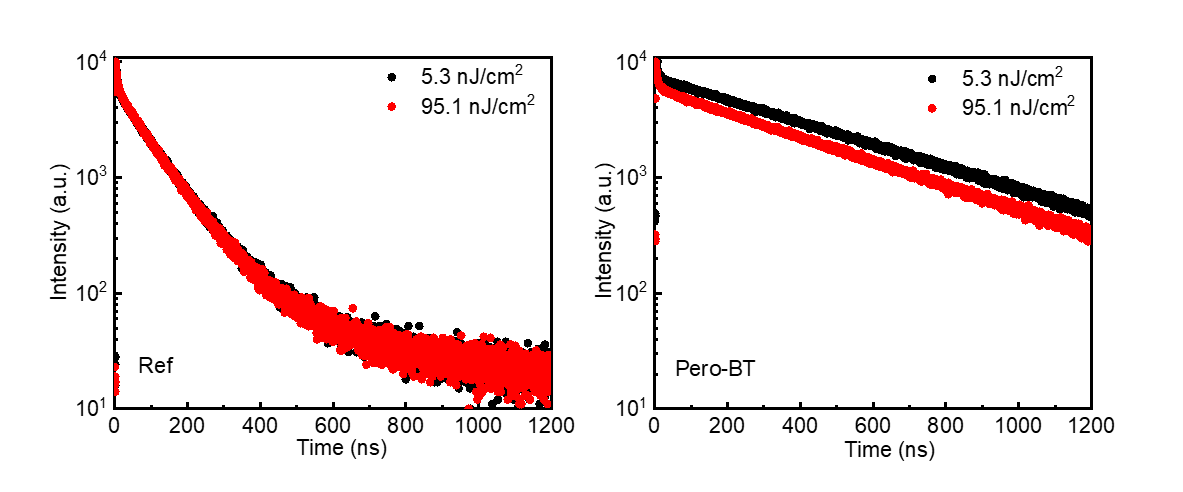


Figure S14. Transient PL decay time of perovskite wi/wo VP-GBA treatment processing under different laser intensity excitation.


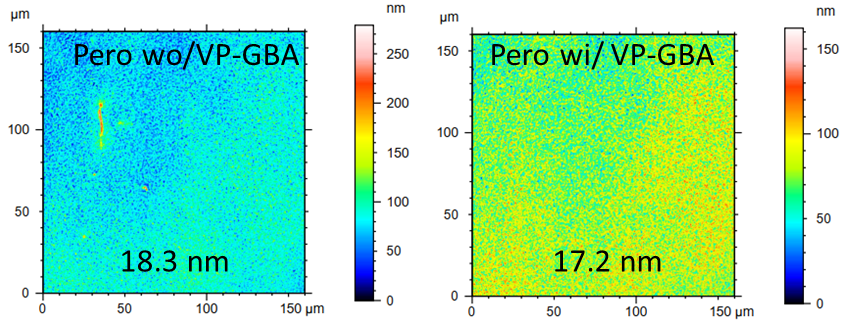


Figure S15. Roughness of topological view of perovskite film is measured by confocal microscopy.


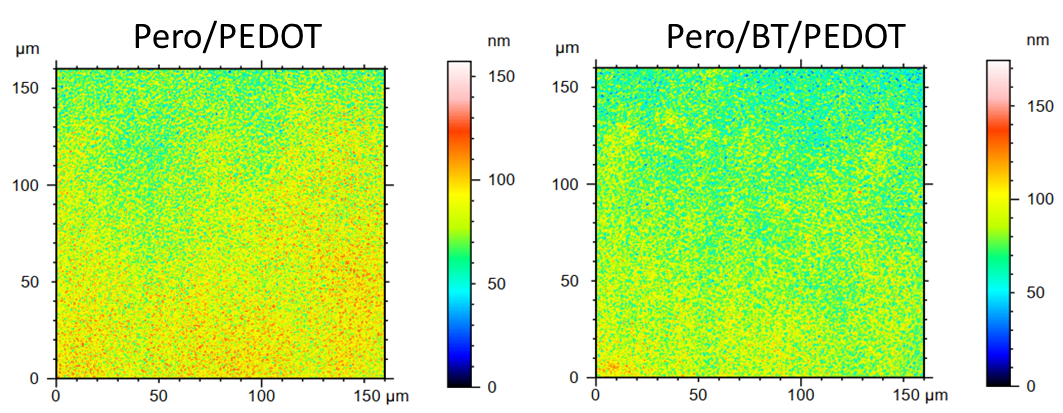


Figure S16. The topological view of perovskite film with HTL on top is measured by confocal microscopy.


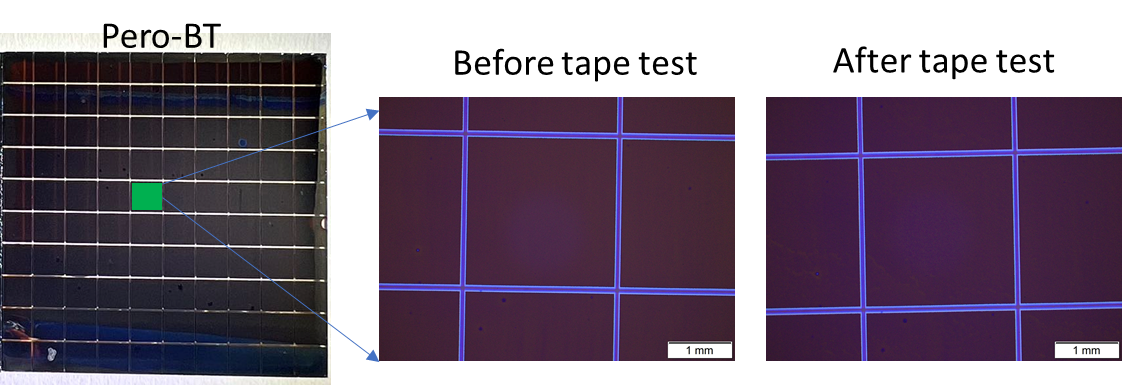


Figure S17. Patterned perovskite film with 100 grids carried out with sticky tape test repeatedly and the optical photograph of perovskite before and after tape test.


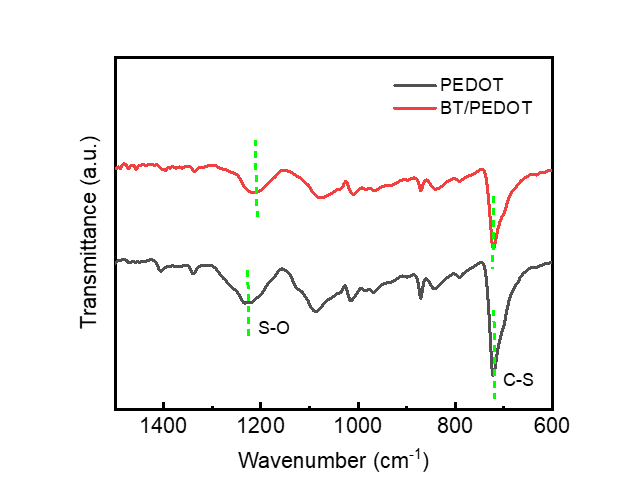


Figure S18. Infrared transmittance spectra of PEDOT on perovskite wi/wo VP-GBA treatment.


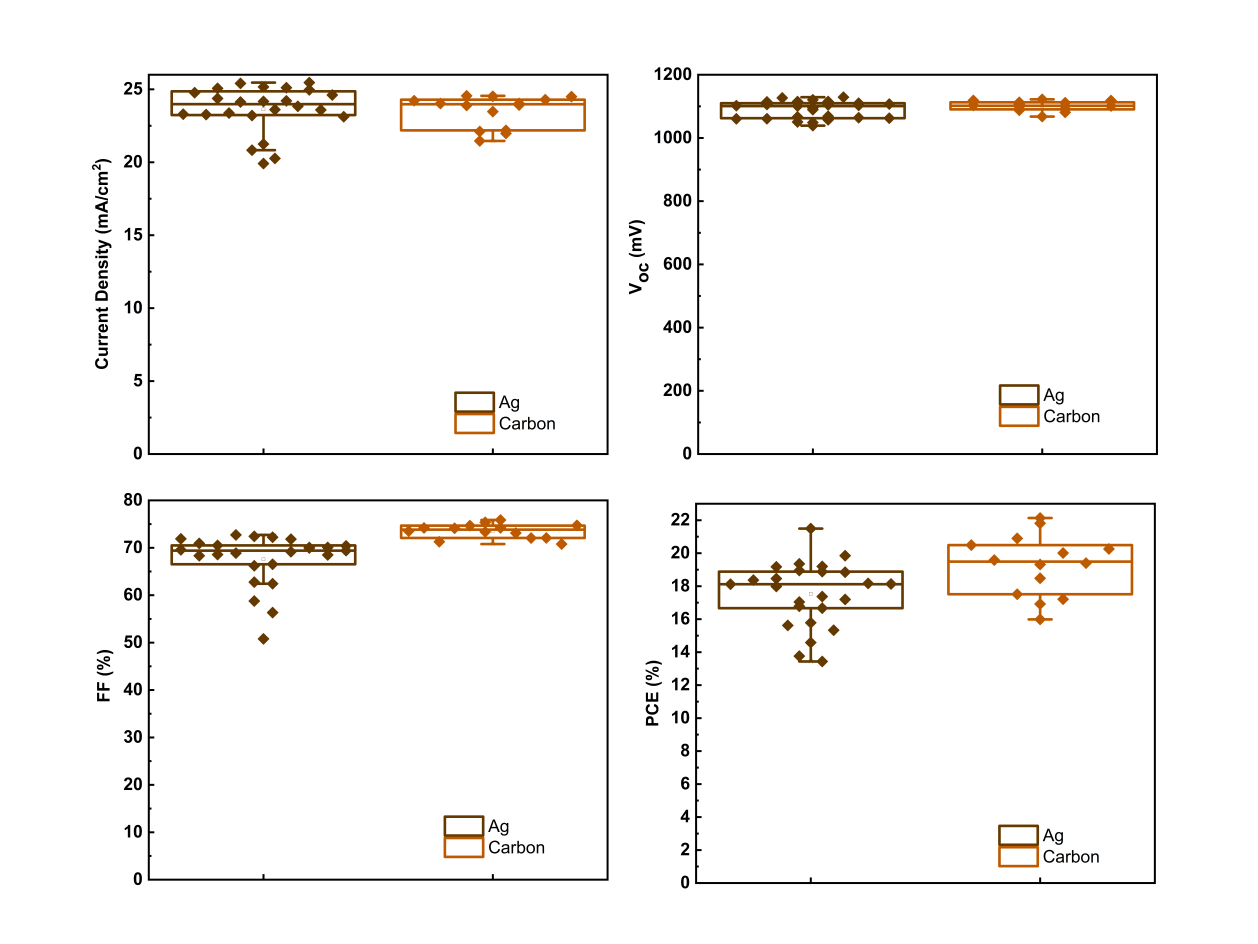


Figure S19. The photovoltaic parameters statistics of R2R printed flexible perovskite solar cells without VP-GBA treatment.


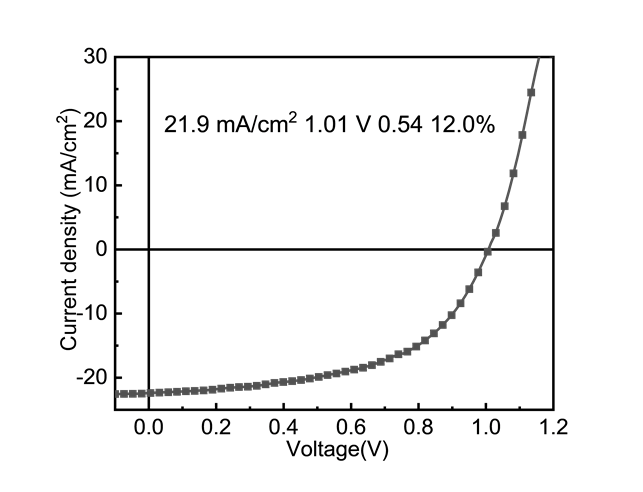


Figure S20. The IV curve reference for R2R printed flexible perovskite solar cells without VP-GBA treatment.


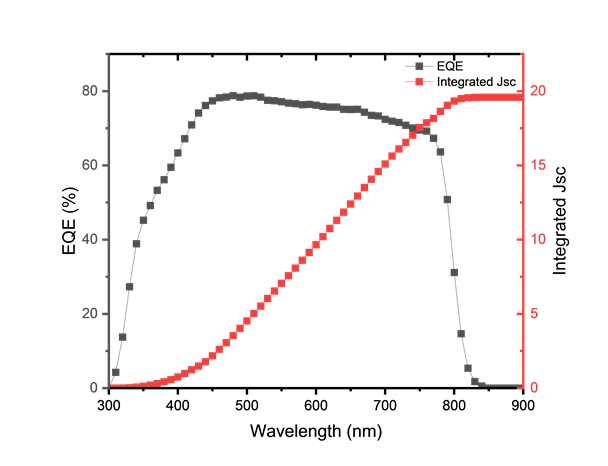


Figure S21. External quantum efficiency of the complete flexible device and corresponding integrated photocurrent of 19.9 mA/cm^2^.


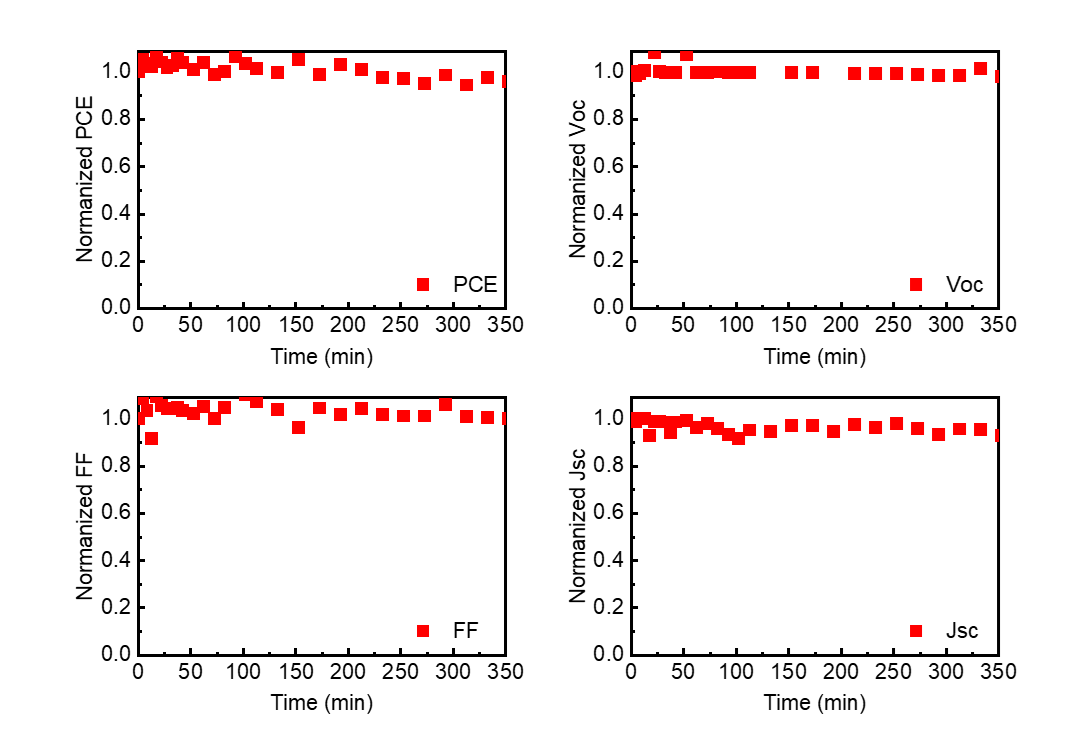


Figure S22. Stabilized power output (SPO) and current of the champion flexible device under maximum power point tracking in 350 mins.


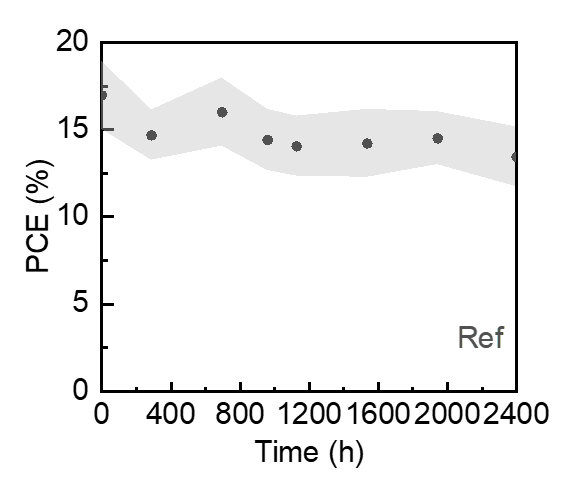


Figure S23. Shelf-lifetime of the reference devices with error band included in ambient environment test with a 30% RH.


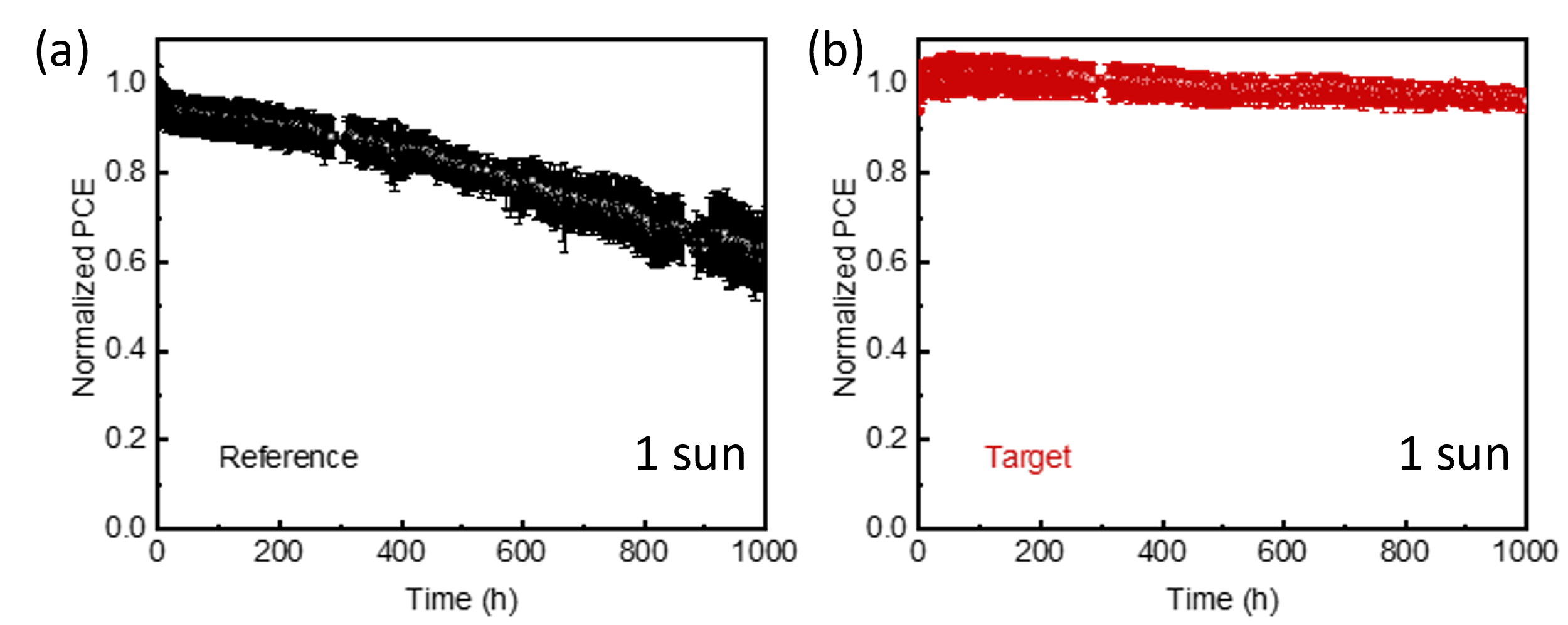


Figure S24: Operational stability of flexible devices (a) without and (b) with BT vapor treatment under continuous 1 sun illumination in nitrogen atmosphere following the ISOS-L-1 protocol.


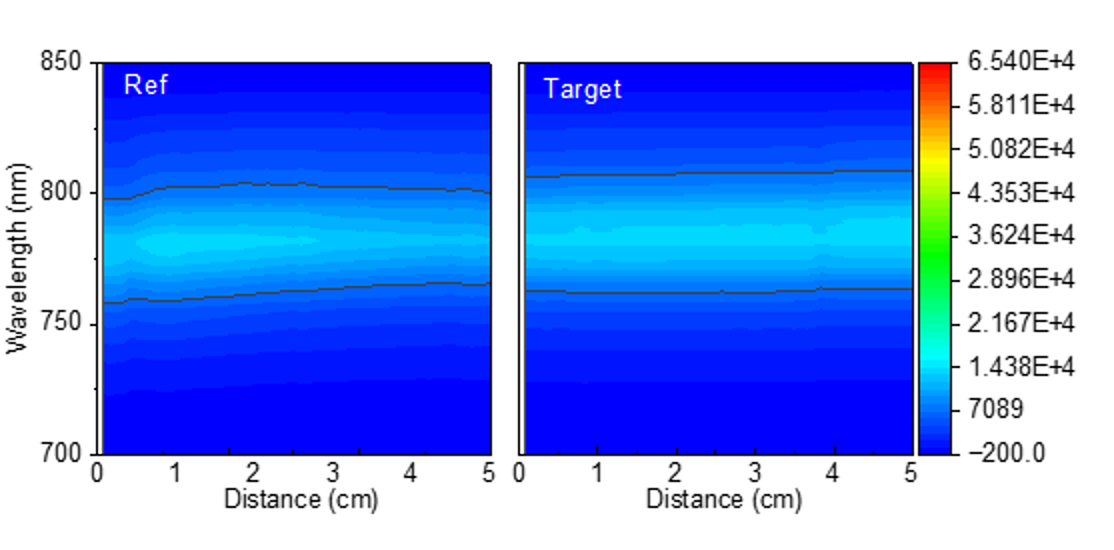


Figure S25. PL mapping of perovskite wi/wo BT vapor treatment on a 5 cm$\times$5 cm size.


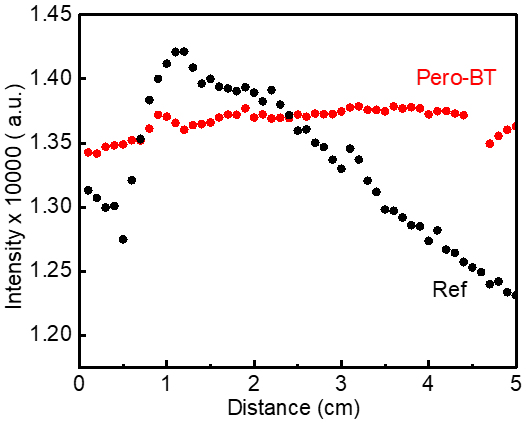


Figure S26. PL intensity of perovskite at wavelength of 781 nm extracted from PL mapping from coating start to end edge.


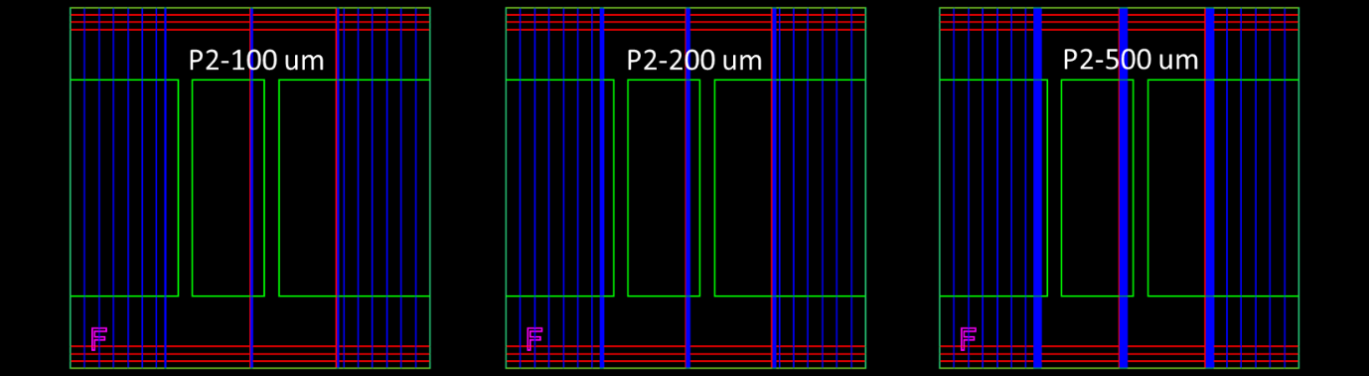


Figure S27. Dimension and design of minimodules with varied P2 width from 100 um to 500 um. The red line is P1, the blue line is P2 and the green line is P3.


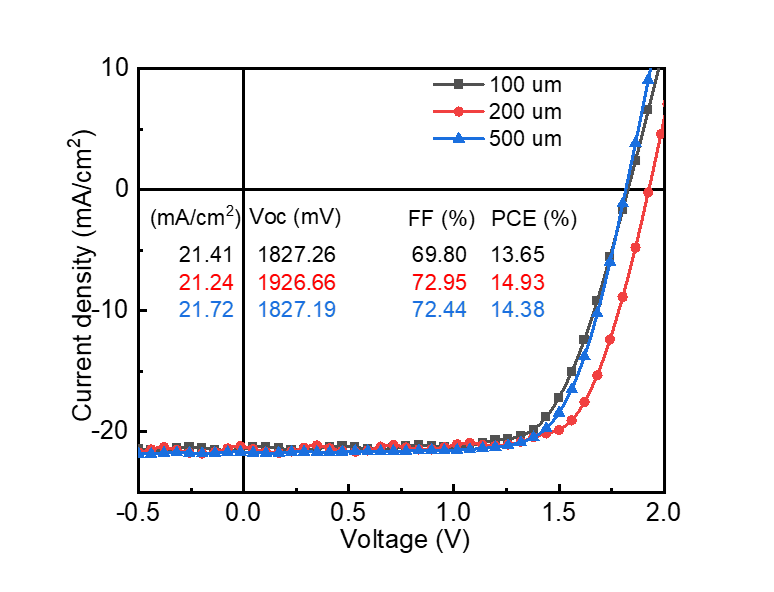


Figure S28. Variation of module photovoltaic efficiency when P2 width is 100 μm, 200 μm, and 500 μm without including GFF (67%).


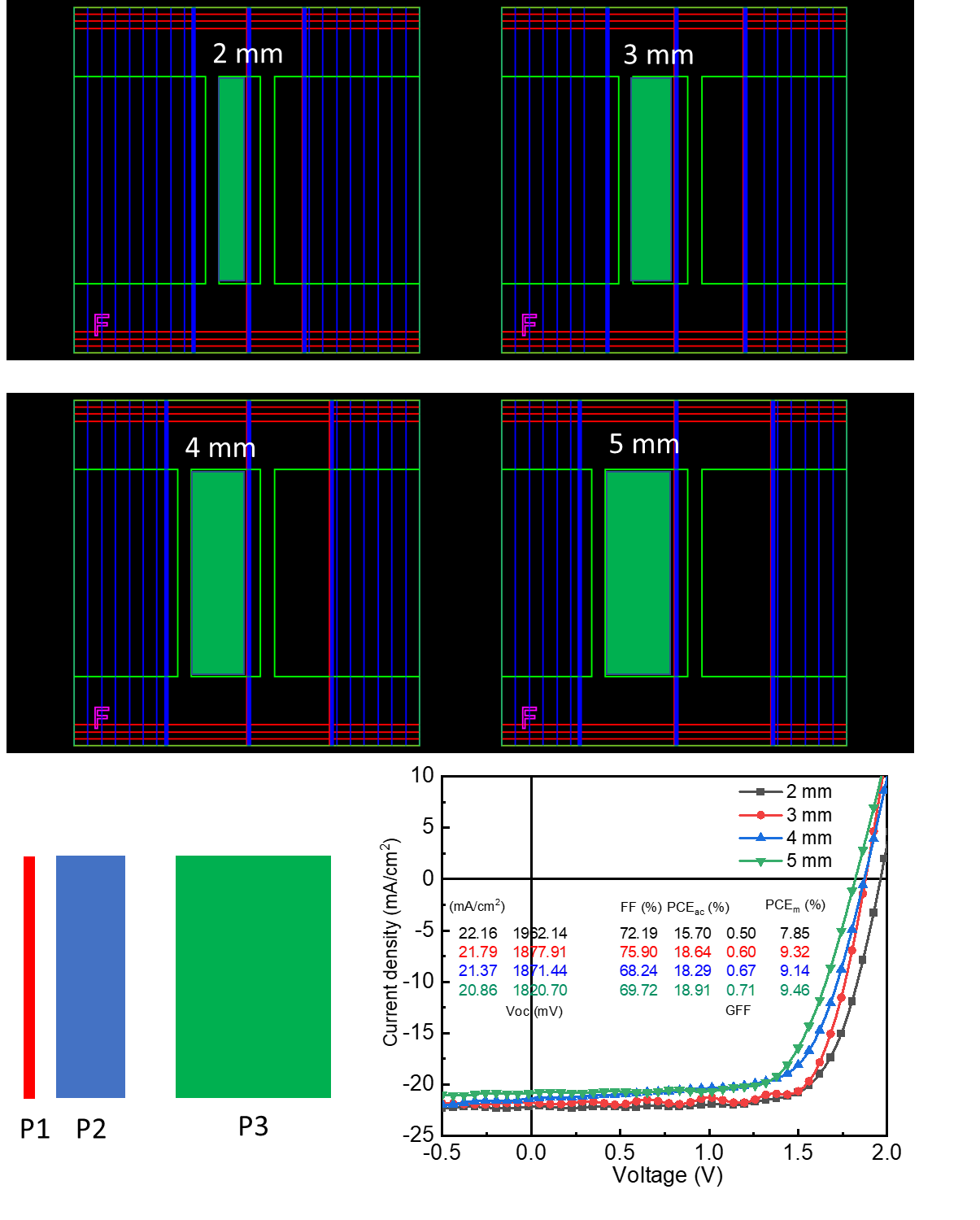


Figure S29. Variation of module photovoltaic efficiency when cell width is 2 mm, 3 mm, 4 mm, and 5 mm. In the module design, three typical scribing lines are defined: P1 (red), P2 (blue), and P3 (green).


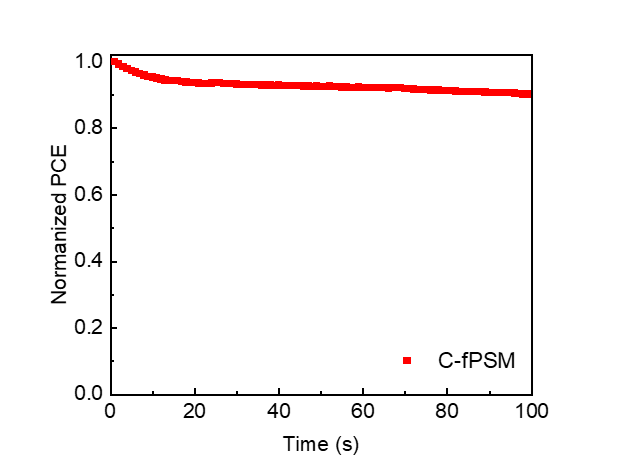


Figure S30. Stabilized power output (SPO) of the champion flexible module under maximum power point tracking in 100 s.


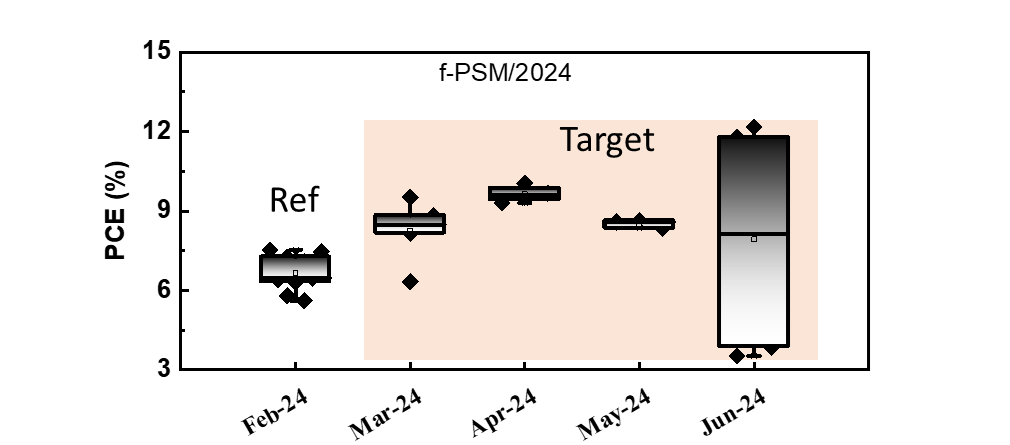


Figure S31. A statistic performance of 5 cm $\times$ 5 cm flexible modules from batch to batch.


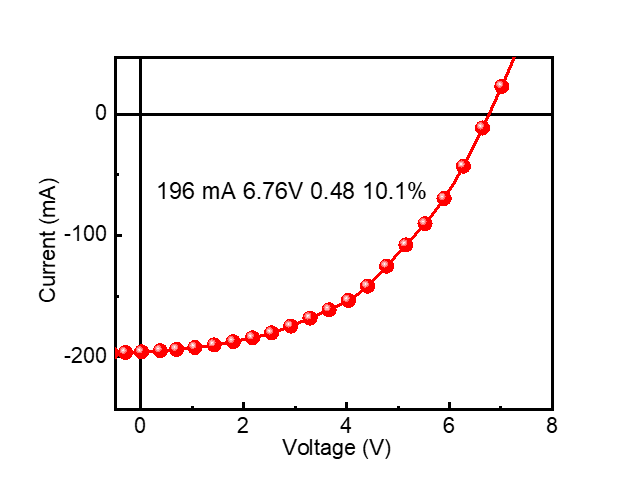


Figure S32. I-V curve of the 100 cm^2^ flexible module with carbon electrode.

Table S1: Comparison of safety and hazards of common solvents and the solvents used in this work.


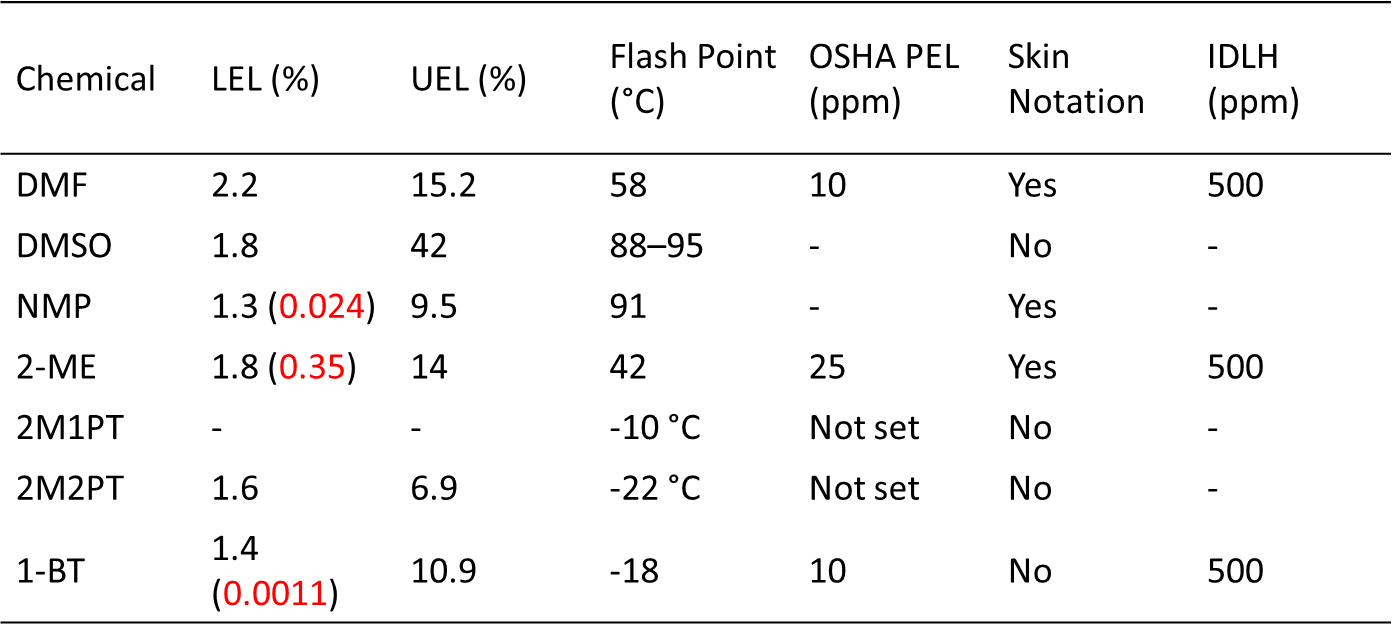


The numbers in red are the concentrations we used in this work.

Notes: data from U.S. National Library of Medicine (PubChem)

LEL (Lower Explosive Limit) and UEL (Upper Explosive Limit) indicate the concentration range in air within which the substance can ignite or explode.

Flash Point is the lowest temperature at which the substance can vaporize to form an ignitable mixture in air.

OSHA PEL (Permissible Exposure Limit) is the maximum amount or concentration of a chemical that a worker may be exposed to under OSHA regulations.

Skin Notation indicates that dermal exposure is a significant route of exposure.

IDLH (Immediately Dangerous to Life or Health) is the concentration that poses a threat of exposure to airborne contaminants when that exposure is likely to cause death or immediate or delayed permanent adverse health effects.

DMF: Dimethylformamide, DMSO: Dimethyl Sulfoxide, NMP: N-Methyl pyrrolidone,2-ME: 2-Methoxyethanol, BT: 1-Butanethiol, 2M1PT: 2-methyl-1-propanethiol, 2M2PT: 2-methyl-2-propanethiol

Table S2: Atomic composition of S and Pb with different etching times.

| **Etch time (s)** | **S (at.%)** | **Pb (at.%)** |
| --- | --- | --- |
| 0 | 9.35 | 90.65 |
| 60 | 9.12 | 90.88 |
| 120 | 8.71 | 91.29 |
| 180 | 8.4 | 91.6 |
| 240 | 8.85 | 91.15 |
| 300 | 8.63 | 91.37 |

Table S3: Average Hardness and Modulus and stardard deviations (std) of perovskite film wi/wo BT vapor treatments.


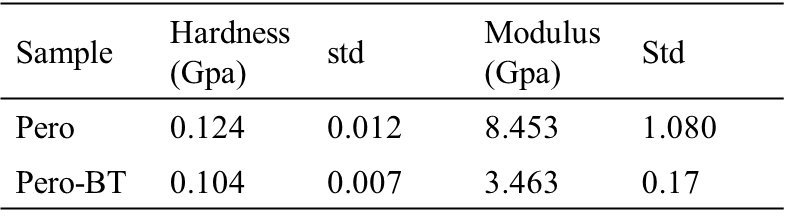


Note: The values for Hardness and Modulus are means with std.

Table S4. Statistical comparison of mechanical stability for flexible perovskite devices reported in recent literature.


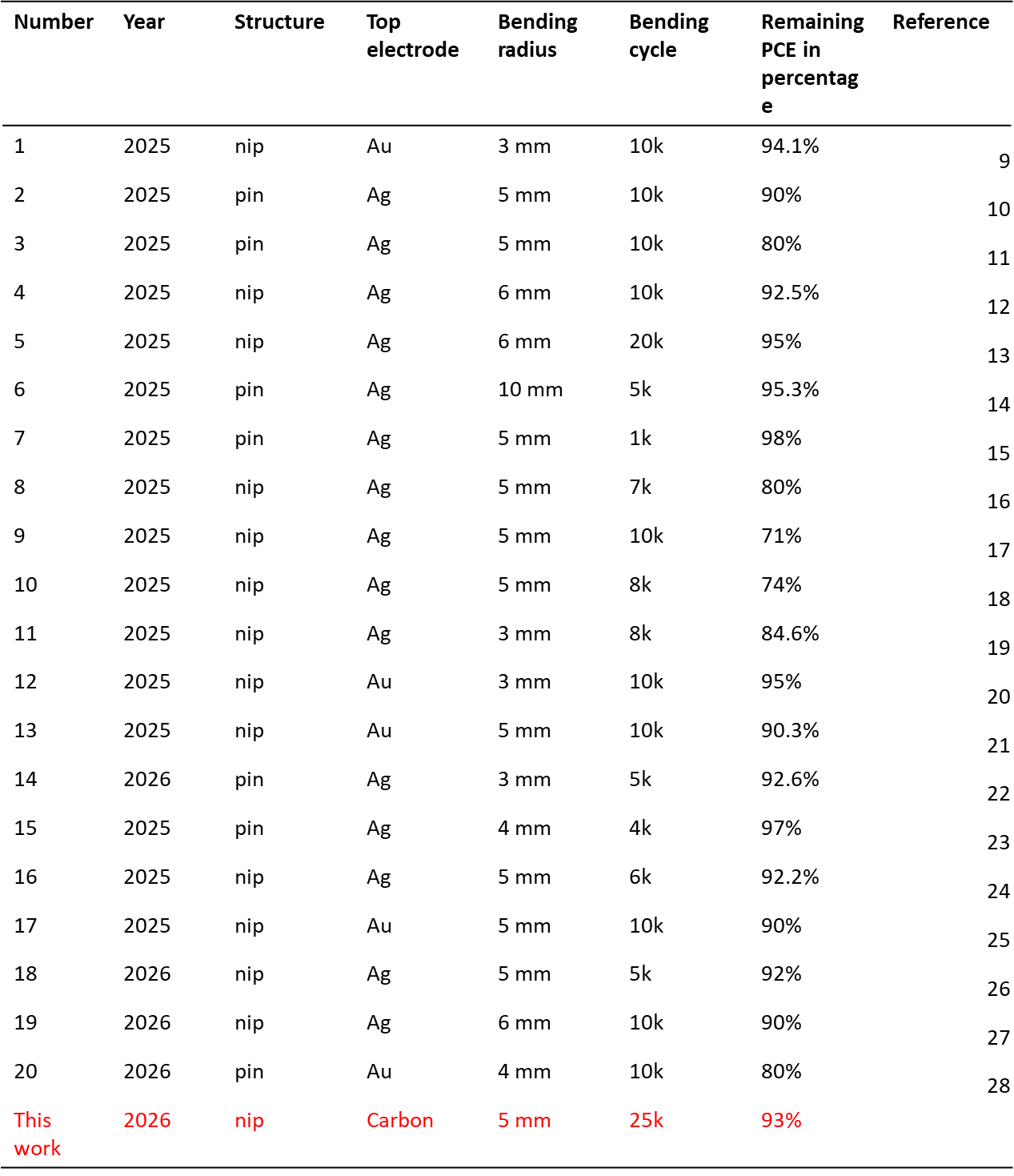


**References**:

[1] G. Kresse, J. Hafner, Ab Initio molecular Dynamics for Liquid Metals, Phys. Rev. B 47 (1993) 558-561.

[2] G. Kresse, J. Hafner, Ab initio molecular-dynamics simulation of the liquid-metalamorphous-semiconductor transition in germanium, Phys. Rev. B Condens. Matter Mater. Phys. 49 (1994) 14251–14269.

[3] J. P. Perdew, K. Burke, M. Ernzerhof, Generalized Gradient Approximation Made Simple. Phys. Rev. Lett. 77 (1996) 3865-3868.

[4] G. Kresse, D. Joubert, From ultrasoft pseudopotentials to the projector augmentedwave method, Phys. Rev. B Condens. Matter Mater. Phys. 59 (1999) 1758–1775.

[5] S. Grimme, J. Antony, S. Ehrlich, H.A. Krieg, Consistent and accurate ab initio parametrization of density functional dispersion correction (DFT-D) for the 94 elements H-Pu, J. Chem. Phys. 132 (2010), 154104.

[6] K. Momma and F. Izumi, J. Appl. Crystallogr., 44, 1272-1276 (2011).

[7] W. C. Oliver and G. M. Pharr, Journal of Materials Research, 1992, 7, 1564-1583.

[8] Robin Bendall, M., and Gordon, R.E. (1983). J. Magn. Reson. 53, 365–385.

[9] Li Z, Jia C, Wan Z, et al. Boosting mechanical durability under high humidity by bioinspired multisite polymer for high-efficiency flexible perovskite solar cells[J]. Nature Communications, 2025, 16(1): 1771.

[10] Zhang W, Liu J, Song W, et al. Chemical passivation and grain-boundary manipulation via in situ cross-linking strategy for scalable flexible perovskite solar cells[J]. Science Advances, 2025, 11(5): eadr2290.

[11] Xu Z, Yu R, Xue T, et al. Stress release via thermodynamic regulation towards efficient flexible perovskite solar cells[J]. Energy & Environmental Science, 2025, 18(9): 4324-4334.

[12] Ning L, Yao Z, Zha L, et al. High‐Oriented SnO2 Nanocrystals for Air‐Processed Flexible Perovskite Solar Cells with an Efficiency of 23.87%[J]. Advanced Materials, 2025, 37(27): 2418791.

[13] Yang Z, Wei J, Liu Y, et al. Radical p‐Doping Spiro‐OMeTAD for Efficient, Stable and Self‐Healing Flexible Perovskite Solar Cells[J]. Advanced Materials, 2025, 37(27): 2417404.

[14] Liang H, Zhu W, Lin Z, et al. Enhancing Efficiency and Stability of Inverted Flexible Perovskite Solar Cells via Multi‐Functionalized Molecular Design[J]. Angewandte Chemie International Edition, 2025, 64(24): e202501267.

[15] Rabehi I N, Mariotti S, Fukuda K, et al. Dual hole transport layer for ultra-flexible perovskite solar cells with unprecedented stability[J]. Joule, 2025, 9(12).

[16] Xu Z, Yu R, Lv Q, et al. Tensile strain regulation via grain boundary buffering for flexible perovskite solar cells[J]. Nature Communications, 2025.

[17] Xu Y, Zhang S, Yuan H, et al. Highly efficient and stable flexible perovskite solar cells enabled by alkylammonium acetate modification with varied dipole moments[J]. Advanced Functional Materials, 2025, 35(23): 2422014.

[18] Xu Y, Zhang S, Yuan H, et al. Mechanically resilient and highly efficient flexible perovskite solar cells with octylammonium acetate for surface adhesion and stress relief[J]. ACS nano, 2025, 19(4): 4867-4875.

[19] Luo X, Zhong Y, Gao B, et al. Dissected MACl Involved Reaction Pathway for Low Temperature Fabrication of High‐Efficiency Flexible Perovskite Solar Cells[J]. Angewandte Chemie, 2025, 137(31): e202502949.

[20] Li Z, Jia C, Wu H, et al. In‐Situ Cross‐Linked Polymers for Enhanced Thermal Cycling Stability in Flexible Perovskite Solar Cells[J]. Angewandte Chemie, 2025, 137(10): e202421063.

[21] Liu W, Xu G, Wu Y, et al. Self‐Healing Hydrophobic Buried Interfaces for Achieving Moisture‐Resistant Flexible Perovskite Solar Cells with 26.38% Efficiency[J]. Advanced Materials, 2025: e19163.

[22] Sun X, Gong J, Liu Q, et al. Molecular Tailoring of Self‐Assembled Monolayers via Polar Ether Linker for Highly Efficient and Mechanically Robust Flexible Perovskite Solar Cells[J]. Advanced Materials, 2026, 38(7): e19365.

[23] Zhou B, Li M, Xiong Q, et al. Soft conjugation extension strategy of self-assembled molecules for achieving efficient and mechanically stable flexible perovskite solar cells[J]. Energy & Environmental Science, 2025, 18(19): 8803-8814.

[24] Duan M, Yang J, Li T, et al. Mechanically stable screen-printed flexible perovskite solar cells via selective self-assembled siloxane coupling agents[J]. npj Flexible Electronics, 2025, 9(1): 30.

[25] Zheng H, Liu G, Dong X, et al. Self-regulated bilateral anchoring enables efficient charge transport pathways for high-performance rigid and flexible perovskite solar cells[J]. Nano-Micro Letters, 2025, 17(1): 328.

[26] Zhao L, Zhang J, Hua Y, et al. Chemical bridging in a 2D/3D heterojunction via dual-anchoring functionalized molecules for efficient, stable and flexible perovskite solar cells[J]. Energy & Environmental Science, 2026.

[27] Shi C, Zheng X, Duan R, et al. Modulate Stresses for Efficient Full‐Air Processed Flexible Perovskite Solar Cells with Polymer Adhesive[J]. Small, 2026, 22(6): e12250.

[28] Wang C, Zhang C, Wang Q, et al. Co‐Self‐Assembled Interface Engineering Assisted for Bend‐Resistant and Efficient Flexible Perovskite Solar Cells[J]. Advanced Science, 2026, 13(3): e09724.
